# Immune Checkpoint Inhibitor Therapy and Risk of Glomerular Disease: A Large Propensity-Matched Cohort Study

**DOI:** 10.3390/life16091576

**Published:** 2026-09-21

**Authors:** Ping-Huang Tsai, Wei-Cheng Chang, Hong-Jie Jhou, Hsin-Yu Chen, Li-Ting Kao, Tina Yi-Jin Hsieh, Po-Huang Chen, Cho-Hao Lee

**Affiliations:** 1 Molecular Immunity Unit, Department of Medicine, University of Cambridge, MRC Laboratory of Molecular Biology, Cambridge CB2 0QQ, UK; pht32@cam.ac.uk; 2 Cambridge Institute of Therapeutic Immunology and Infectious Diseases, University of Cambridge, Cambridge CB2 0AW, UK; 3 Division of Nephrology, Department of Internal Medicine, Tri-Service General Hospital, National Defense Medical University, Taipei 114202, Taiwan; 4 Department of Ophthalmology, Universe Eye Center, Keelung 20041, Taiwan; cwc761229@gmail.com; 5 Neurological Institute, Changhua Christian Hospital, Changhua 50006, Taiwan; xsai4295@gmail.com; 6 Department of Family Medicine, Chi Mei Medical Center, Tainan 71004, Taiwan; yupucca@gmail.com; 7 Graduate Institute of Life Sciences, National Defense Medical University, Taipei 114201, Taiwan; kaoliting@office365.ndmctsgh.edu.tw; 8 School of Pharmacy, National Defense Medical University, Taipei 114201, Taiwan; 9 Department of Pharmacy Practice, Tri-Service General Hospital, Taipei 114202, Taiwan; 10 Department of Obstetrics & Gynecology, Beth Israel Deaconess Medical Center, Boston, MA 02215, USA; hsiehyijin@gmail.com; 11 Division of Hematology and Oncology, Department of Internal Medicine, Tri-Service General Hospital, National Defense Medical University, Taipei 114202, Taiwan; 12 Department of Oncology, Tri-Service General Hospital, National Defense Medical University, Taipei 114202, Taiwan

**Keywords:** cancer treatment, immunotherapy, clinical outcomes, onco-nephrology, glomerular disease

## Abstract

Immune checkpoint inhibitors (ICIs) have transformed cancer treatment but may cause immune-related kidney injury, and their association with glomerular disease remains incompletely understood. We evaluated the risk of incident glomerular disease following ICI therapy using the TriNetX U.S. Collaborative Network. Adult patients with cancer treated between January 2014 and March 2025 who received ICIs were compared with those receiving conventional antineoplastic therapies after 1:1 propensity score matching for demographics, cancer type, comorbidities, medications, and baseline kidney function. The index date was constrained to an eligibility window ending 1 March 2024, and identical exposure requirements were applied to both cohorts. The primary outcome was incident glomerular disease (ICD-10-CM codes N00, N01, N02, N04, N05 and N08), and hazard ratios (HRs) were estimated using Cox proportional hazards models. The proportional hazards assumption was tested for every outcome, death was additionally modelled as a competing event, and subgroup heterogeneity was assessed by formal tests of interaction. Among 71,354 matched patient pairs, glomerular disease occurred in 2.5% of ICI-treated patients and in 2.4% of controls. ICI therapy was associated with a higher hazard of glomerular disease (HR 1.492, 95% CI 1.392–1.599). The excess hazard was concentrated in the first 500 days after initiation (interval HR 1.68); beyond approximately 1000 days, the interval point estimates approached unity. In a supporting analysis in which death was modelled as a competing event, the estimated 5-year cumulative incidence was 4.5% with ICI therapy and 3.2% with comparator therapy, an absolute difference of approximately 1.2 percentage points, and the corresponding difference in restricted mean time free of glomerular disease was about 20 days. These absolute estimates were derived from the survival functions of the matched cohort rather than from patient-level competing-risk regression and should be read as approximations. In exploratory analyses, heterogeneity was demonstrated by race, cancer type and ICI agent. Renal biopsy and urine protein testing were performed at indistinguishable rates in the two cohorts, arguing against differential detection. ICI therapy is associated with a modest absolute increase in the risk of coded incident glomerular disease, concentrated in the first 18 months, which may support targeted rather than indefinite renal surveillance.

## 1. Introduction

Immune checkpoint inhibitors (ICIs) have transformed cancer therapy by enhancing the immune system’s ability to recognize and eliminate malignant cells through the blockade of inhibitory signalling pathways [1]. Since the first approval of ipilimumab in 2011, ICIs targeting cytotoxic T-lymphocyte-associated protein 4 (CTLA-4), programmed death-1 (PD-1), and programmed death-ligand 1 (PD-L1) have become standard treatments for a broad range of malignancies, including melanoma, non-small-cell lung cancer, and renal cell carcinoma [2]. However, their widespread adoption has revealed a broad spectrum of immune-related adverse events (irAEs) involving the skin, gastrointestinal tract, endocrine organs, and, notably, the kidneys [3]. Among renal toxicities, acute interstitial nephritis is well-documented, but the risk and characteristics of glomerular disease remain poorly understood and likely underrecognized.

ICI-associated nephrotoxicity has been reported in 2% to 17% of treated patients, with acute kidney injury (AKI) as the most recognized clinical manifestation [4,5]. Although acute tubulointerstitial nephritis remains the predominant histopathologic finding—accounting for 70–90% of cases—emerging evidence points to a broader spectrum of renal involvement beyond the tubulointerstitial compartment [6]. Case reports and small series have increasingly documented glomerular diseases associated with ICI therapy, including minimal change disease, membranous nephropathy, focal segmental glomerulosclerosis, and immune complex glomerulonephritis [7,8]. A recent review of these reports concluded that podocytopathies and pauci-immune glomerulonephritis predominate but that the incidence relative to other antineoplastic therapies remains unquantified [9].

Despite growing recognition of glomerular involvement in ICI-treated patients, robust epidemiologic data quantifying this risk are limited. Prior studies have predominantly focused on AKI or drawn conclusions from small, uncontrolled case series [10]. Moreover, the comparative risk of glomerular disease between ICI therapy and conventional antineoplastic treatments has yet to be systematically evaluated in large, real-world cohorts.

To address these gaps, we conducted a large, propensity score-matched cohort study to: (1) quantify the risk of glomerular disease associated with ICI therapy relative to conventional antineoplastic agents; (2) identify high-risk patient subgroups; and (3) characterize the timing and outcomes of ICI-associated glomerular disease.

## 2. Materials and Methods

### 2.1. Study Design and Data Source

We conducted a retrospective new-user, active-comparator cohort study within a target trial emulation framework [11], using de-identified electronic health records from the TriNetX U.S. Collaborative Network, a federated multi-institutional database of academic medical centres, community hospitals and specialty practices in the United States; 74 healthcare organizations were queried and 74 responded. The network employs standardized data mapping and quality assurance protocols to ensure data consistency and completeness across participating institutions. The study period spanned from 1 January 2014 to 1 March 2025, coinciding with the widespread clinical adoption of immune checkpoint inhibitors. Patients were eligible to enter a cohort only if the index treatment was first administered between 1 January 2014 and 1 March 2024, so that every patient had at least 12 months of potential follow-up; this constraint was applied identically to both cohorts (Supplementary Table S1). The study protocol was approved by the Institutional Review Board (TSGHIRB No.: E202516013), and informed consent was waived due to the use of de-identified data.

### 2.2. Study Population

Adults aged ≥18 years with a confirmed diagnosis of malignancy who initiated either ICI therapy or conventional antineoplastic treatment were eligible for inclusion. The ICI cohort comprised patients who received any approved ICI agent, including pembrolizumab, nivolumab, atezolizumab, durvalumab, avelumab, ipilimumab, or cemiplimab on at least two occasions; the requirement for at least two administrations was applied symmetrically to the comparator cohort. The comparator cohort consisted of patients who received conventional cytotoxic chemotherapy, tyrosine kinase inhibitors, or non-immune monoclonal antibodies during the same study period. Exclusion criteria were: (1) age < 18 years; (2) absence of a confirmed cancer diagnosis; (3) pre-existing glomerular disease or other significant renal pathology prior to the index date (N00–N08, N18.6 or Z99.2 at any time on or before the first malignancy diagnosis); (4) receipt of any antineoplastic therapy before cancer diagnosis; and (5) administration of both ICI and non-ICI therapies during the initial treatment period. In addition, for every outcome, patients with that outcome recorded before the start of the analysis window were excluded from the analysis of that outcome, so that all estimates refer to incident events. Full code lists are provided in Supplementary Table S2.

### 2.3. Exposure Definition

The index date was defined as the date of first administration of either an ICI agent (for the ICI cohort) or a conventional antineoplastic agent (for the control cohort) occurring within the index eligibility window. The date constraint was applied to the index event definition itself rather than only to the cohort-qualifying criteria, so that patients could not qualify for a cohort on the basis of treatment administered after their assigned time origin [12]. Exposure to ICI therapy was identified using RxNorm codes corresponding to approved agents. Nephrotoxic chemotherapeutic agents, including cisplatin, carboplatin, oxaliplatin, ifosfamide, cyclophosphamide, melphalan, carmustine, and methotrexate, were systematically identified and monitored for use in sensitivity analyses (Supplementary Table S3).

### 2.4. Outcomes

The primary outcome was new-onset glomerular disease, defined as a composite outcome based on ICD-10 diagnostic codes, including acute nephritic syndrome (N00), rapidly progressive nephritic syndrome (N01), recurrent and persistent haematuria (N02), nephrotic syndrome (N04), unspecified nephritic syndrome (N05), and glomerular disorders in diseases classified elsewhere (N08). Proteinuria (R80) was not included in the primary composite because it is a sign rather than a glomerular disease diagnosis; it was analysed separately. N03, N06 and N07 were exclusion criteria rather than outcome components. Secondary outcomes included individual glomerular disease subtypes (acute nephritic syndrome, N00 and N01; nephrotic syndrome, N04), acute kidney injury (N17), progression of chronic kidney disease (N18.1–N18.5), tubulointerstitial nephritis (N12, N14.1 and N14.2), proteinuria (R80), haematuria (R31), and clinically significant electrolyte imbalances (E87). Because diagnostic codes cannot distinguish biopsy-confirmed from clinically suspected disease, we additionally examined a high-specificity definition restricted to N00, N01 and N04. Complete outcome definitions are given in Supplementary Tables S4 and S5.

### 2.5. Propensity Score Matching

To mitigate confounding by indication and baseline imbalances between treatment groups, 1:1 propensity score matching was performed using a greedy nearest-neighbour algorithm without replacement. The propensity score was estimated via multivariable logistic regression incorporating forty covariates spanning all measured baseline covariates, including demographics, cancer type, comorbidities, concomitant medications, and baseline kidney function (Supplementary Tables S7 and S8). A calliper width was set at 0.2 times the standard deviation of the logit of the propensity score. Covariate balance was evaluated using standardized mean differences (SMDs), with values < 0.1 considered indicative of adequate balance [13].

### 2.6. Statistical Analysis

The primary analysis employed Cox proportional hazards regression to estimate hazard ratios and 95% confidence intervals for the risk of glomerular disease, with robust standard errors accounting for the matched-pair structure. Patients were followed from the index date until the first occurrence of the outcome, death, or the end of the study period (1 March 2025), whichever came first. The proportional hazards assumption was assessed for every outcome. Where it was violated, the hazard ratio is reported as a time-averaged effect and is supplemented by two analyses that do not require the assumption: restricted mean survival time (RMST) [14] at 1, 3 and 5 years, and a non-overlapping interval analysis in which each interval counts only the hazard accrued within it among patients still under observation at its start. Because death is a frequent competing event in an oncology population, cumulative incidence was additionally estimated using the Aalen–Johansen estimator with death as a competing event [15]; cause-specific cumulative hazards for glomerular disease and for death were obtained from the corresponding Kaplan–Meier estimates in the matched cohort. Absolute risk differences and the number needed to harm were derived from these cumulative incidence functions. E-values were calculated for the primary estimate and its lower confidence limit [16]. Sensitivity analyses included: (1) an on-treatment analysis requiring at least six treatment administrations within the first six months, applied symmetrically to both cohorts, with the outcome window beginning at day 180; (2) exclusion of patients receiving nephrotoxic chemotherapy; (3) exclusion of ICI patients who subsequently received nephrotoxic chemotherapy; and (4) landmark analyses excluding events occurring within 30, 90, and 180 days after the index date. Subgroup analyses were stratified by age, sex, race/ethnicity, baseline kidney function, cancer type, specific ICI agent, and concomitant medications. Because differences between subgroup estimates do not by themselves indicate effect modification [17], heterogeneity across the strata of each subgroup domain was tested formally by Cochran Q on the log hazard ratio scale, with one test per domain and Benjamini–Hochberg adjustment across domains [18]; pairwise contrasts are post hoc. Subgroup analyses were not prespecified in a statistical analysis plan, and matching was not repeated within strata, so all subgroup findings are reported as exploratory. Negative control outcomes, including appendicitis, suicide attempts, and burns, were analysed to evaluate residual confounding [19], and two positive control outcomes representing established immune-related adverse events (thyroid dysfunction and drug-induced colitis) were analysed to confirm that the analytic pipeline detects true immune-related events (Supplementary Table S6). To assess differential detection, the proportions of patients undergoing renal function testing, urine protein testing and renal biopsy were compared between cohorts. All statistical analyses were performed using R version 4.2.1, with two-sided *p*-values < 0.05 considered statistically significant.

## 3. Results

### 3.1. Study Cohort Characteristics

**Figure 1:**
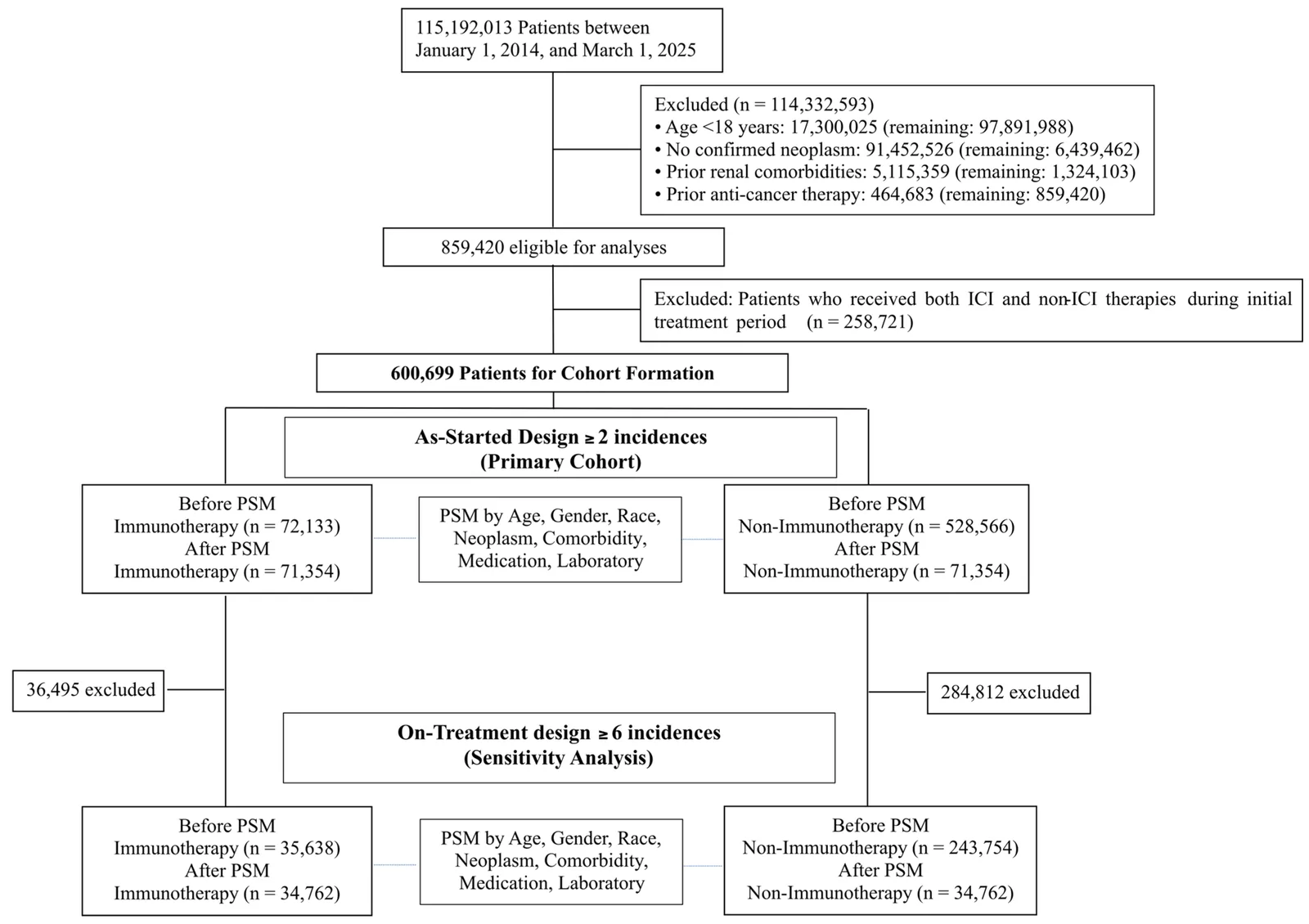
Flow diagram illustrating patient selection, exclusion criteria, and final cohort composition. Among 115,192,013 individuals in the source population, 600,699 eligible patients with cancer were identified. After propensity score matching, 71,354 matched pairs were included in the primary analysis.

After applying exclusion criteria, a total of 600,699 eligible cancer patients were identified from an initial population of 115,192,013. Prior to propensity score matching, the ICI cohort included 72,133 patients, whereas the non-ICI cohort included 528,566 patients. Following 1:1 propensity score matching, 71,354 matched pairs (142,708 patients) were included in the primary analysis (Figure 1). Baseline characteristics were well balanced between the matched cohorts (Table 1, Supplementary Tables S9–S11). The mean age was 65.0 years in the ICI group and 64.6 years in the non-ICI group (SMD = 0.033). The distribution of sex (50.7% vs. 50.4% male), race (74.8% vs. 74.8% White), and baseline comorbidities was similar between groups. Baseline kidney function was also comparable (mean eGFR 82.9 vs. 83.8 mL/min/1.73 m^2^; SMD = 0.028).

### 3.2. Primary Outcome: Glomerular Disease Risk

**Figure 2:**
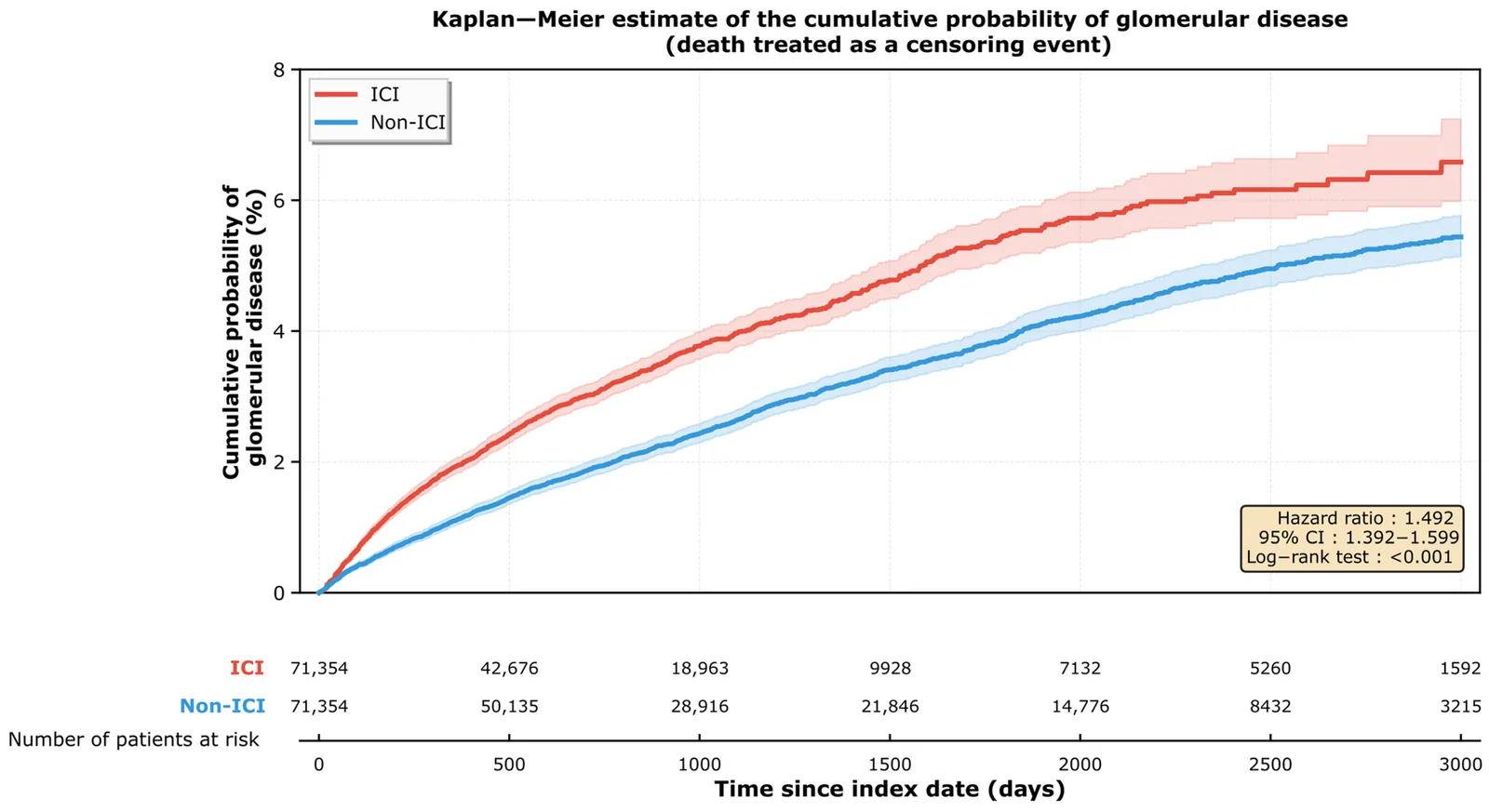
Kaplan–Meier estimate of the cumulative probability of incident glomerular disease among patients with cancer, stratified by immune checkpoint inhibitor (ICI) exposure after propensity score matching (n = 71,354 pairs). Death is treated as a censoring event (hazard ratio 1.492; 95% confidence interval 1.392–1.599; log-rank *p* < 0.001). The curve is a Kaplan–Meier estimate of cumulative probability and not a cumulative incidence function; the corresponding competing-risk analysis, in which death is modelled as a competing event, is reported in Table 2B and Supplementary Table S19. Shaded areas denote 95% confidence intervals, and numbers at risk are shown below the curve.

The median follow-up duration was 764 days (IQR, 1111 days) for the ICI group and 692 days (IQR, 1257 days) for the non-ICI group. During follow-up, glomerular disease occurred in 1784 patients (2.5%) in the ICI group and 1712 patients (2.4%) in the non-ICI group. The overall hazard ratio for glomerular disease was 1.492 (95% CI, 1.392–1.599; *p* < 0.001) (Figure 2), with an E-value of 2.35 for the point estimate and 2.13 for the lower confidence limit. The proportional hazards assumption was violated (*p* < 0.001), so this hazard ratio represents a time-averaged effect and is interpreted alongside the analyses below.

The non-overlapping interval analysis showed that the excess hazard was concentrated early: the interval hazard ratio was 1.68 during days 1–500 and 1.39 during days 501–1000, whereas the point estimates for the subsequent intervals approached unity (1.05, 1.17 and 0.72; Table 2A); however, these interval estimates are not accompanied by confidence intervals and should be read as descriptive. This time course explains the apparent discrepancy between the hazard ratio and the cumulative event proportions. The cumulative risk ratio converges towards the null over longer follow-up because it accumulates person-time during which the two cohorts no longer differ, whereas the hazard ratio, which compares instantaneous rates among patients still at risk, is not diluted in the same way.

Because death is a frequent competing event in this population, and the complement of the Kaplan–Meier function overestimates incidence when competing events are present [15], cumulative incidence was re-estimated with death modelled as a competing event (Table 2B). All-cause mortality was almost identical in the two cohorts (5-year Kaplan–Meier estimate 27.4% versus 26.4%), so the competing risk attenuated the cumulative incidence in both arms by a similar factor, and the corresponding subdistribution hazard ratio was approximately 1.48. At 5 years the cumulative incidence of glomerular disease was 4.47% with ICI therapy and 3.23% with comparator therapy, an absolute difference of 1.24 percentage points, or 12.5 excess cases per 1000 patients treated, corresponding to a number needed to harm of approximately 80. Over the same horizon, patients treated with ICIs spent on average 20.3 fewer days free of glomerular disease (95% CI, 15.9–24.6 days).

### 3.3. Subgroup Analyses

**Figure 3:**
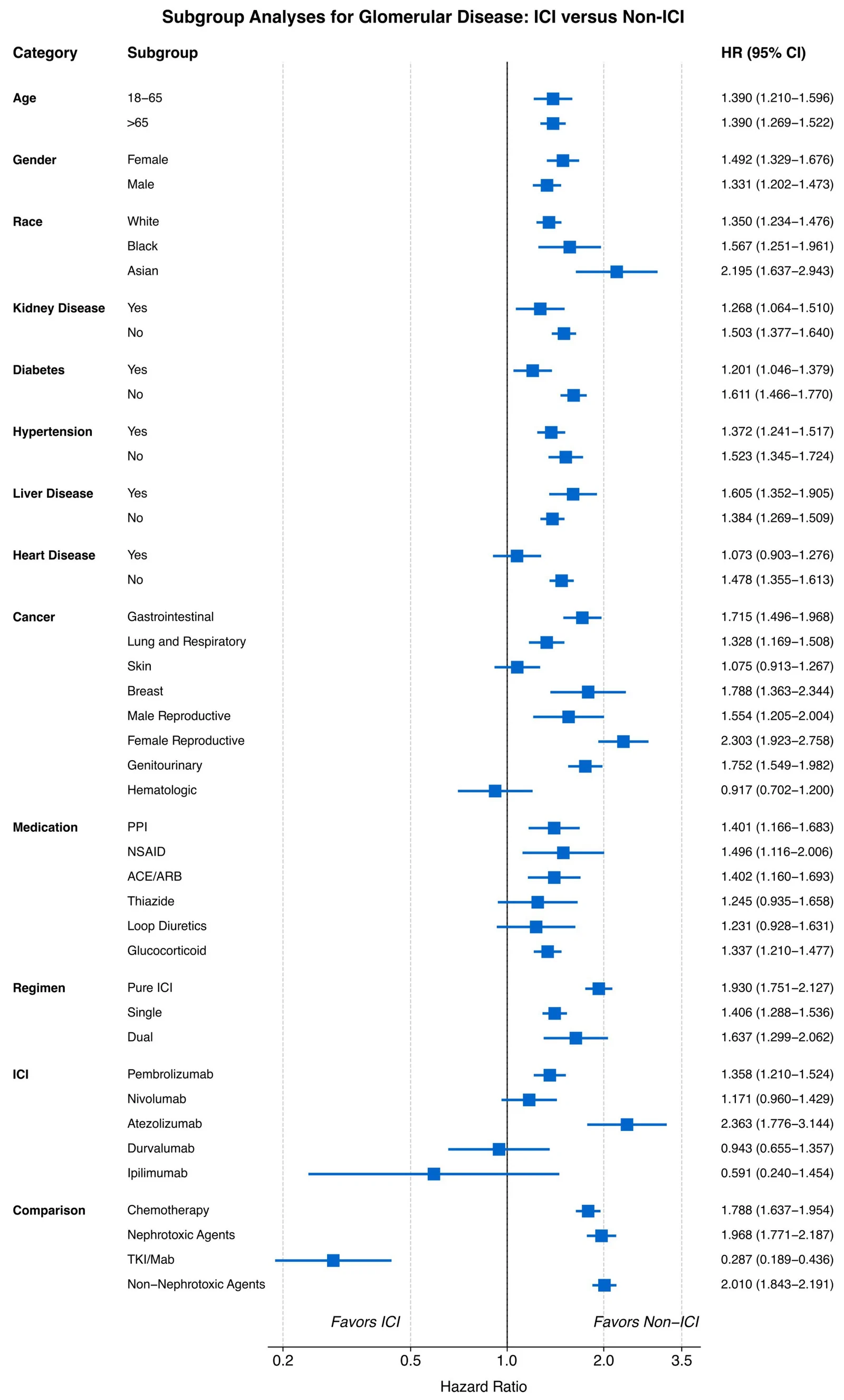
Forest plot showing subgroup analyses of the risk of incident glomerular disease associated with immune checkpoint inhibitor (ICI) therapy. Hazard ratios (HRs) and 95% confidence intervals (CIs) are presented for subgroups defined by demographic characteristics, comorbidities, cancer type, concomitant medications, and treatment regimens in the propensity score-matched cohort. Estimates were derived from Cox proportional hazards models. HRs greater than 1 indicate an increased risk associated with ICI therapy compared with non-ICI treatment. Formal tests of heterogeneity across the strata of each domain, with Benjamini–Hochberg adjustment, are reported in Supplementary Table S18; all subgroup analyses are exploratory.

Subgroup analyses are shown in Figure 3 and formal tests of heterogeneity in Supplementary Table S18. After Benjamini–Hochberg adjustment, heterogeneity was demonstrated for cancer type (q < 0.001), ICI agent (q < 0.001), diabetes mellitus (q = 0.002), ischaemic heart disease (q = 0.003) and race (q = 0.012); the remaining six domains, including age, sex, hypertension, liver disease, baseline kidney disease and single versus dual regimen, were homogeneous (Figure 3). Asian patients exhibited a markedly elevated risk (HR 2.195; 95% CI, 1.637–2.943) compared with White (HR 1.350) and Black patients (HR 1.567); the Asian-versus-White contrast corresponded to a ratio of hazard ratios of 1.63 (95% CI, 1.20–2.21; *p* = 0.002). Patients with female reproductive cancers showed the highest risk (HR 2.303; 95% CI, 1.923–2.758), followed by those with genitourinary (HR 1.752) and gastrointestinal cancers (HR 1.715), and the estimate for female reproductive cancers was significantly higher than that for five of the seven other cancer categories. By contrast, the six concomitant medication subgroups were homogeneous (Q = 1.54, df = 5, *p* = 0.909, I^2^ = 0%), and each confidence interval included the overall estimate, so the numerically higher estimates for proton pump inhibitor, NSAID and ACE inhibitor or ARB users do not indicate effect modification. Two further findings were not anticipated: the association was attenuated in patients with diabetes mellitus (HR 1.201 versus 1.611 without) and in patients with ischaemic heart disease (HR 1.073 versus 1.478 without), both populations in which the background rate of coded renal disease is high. Among ICI agents, atezolizumab demonstrated the highest risk (HR 2.363), significantly higher than pembrolizumab, nivolumab and ipilimumab in post hoc contrasts, whereas ipilimumab monotherapy showed a point estimate below unity with a confidence interval spanning a sixfold range (HR 0.591; 95% CI, 0.240–1.454), reflecting very few events. Dual ICI therapy was associated with a numerically higher estimate than monotherapy (HR 1.637 vs. 1.406), but this difference was not statistically supported (*p* = 0.228). All subgroup analyses are exploratory and hypothesis-generating: they were not prespecified in a statistical analysis plan, and matching was not repeated within strata.

### 3.4. Secondary Renal Outcomes

**Figure 4:**
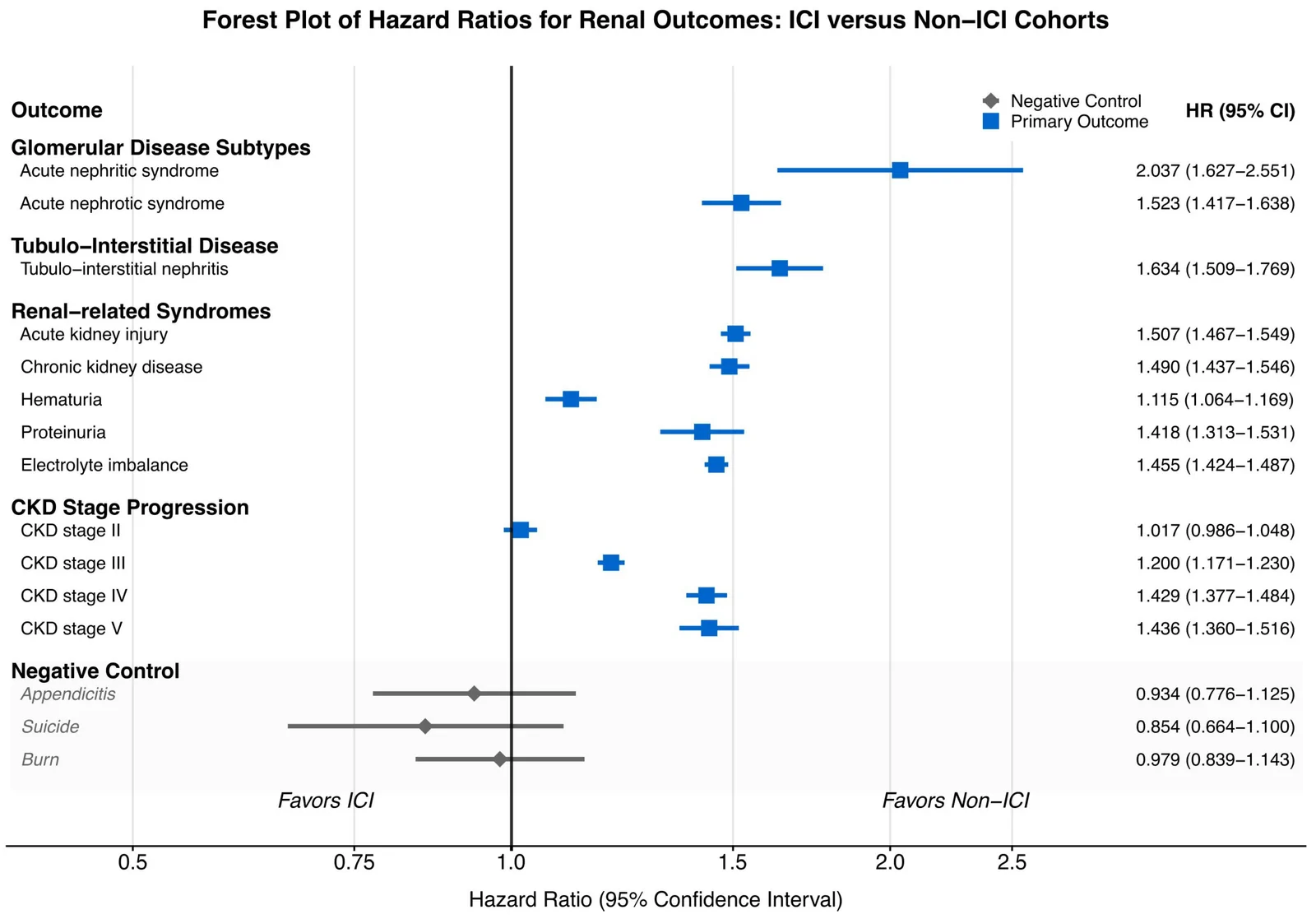
Forest plot comparing adjusted hazard ratios (HRs) and 95% confidence intervals (CIs) for secondary renal outcomes and control outcomes between immune checkpoint inhibitor (ICI) users and non-ICI cohorts. Outcomes include glomerular disease subtypes, tubulo-interstitial disease, renal-related syndromes, and chronic kidney disease (CKD) stage progression. Primary renal outcomes are indicated by blue squares, and negative control outcomes are shown as grey diamonds. The vertical reference line at HR = 1.0 indicates no difference in risk between groups. Negative control outcomes (appendicitis, suicide, and burns) showed no significant association with ICI exposure, supporting the specificity of the observed associations for renal outcomes.

ICI therapy was also associated with elevated risk across several secondary renal outcomes beyond glomerular disease (Figure 4). Acute kidney injury occurred in 17.6% of ICI-treated patients compared with 15.5% in the control group (HR 1.507; 95% CI, 1.467–1.549). Chronic kidney disease developed in 9.5% of patients in both groups but was still associated with a significantly increased risk in the ICI cohort (HR 1.490; 95% CI, 1.437–1.546). Progression to advanced CKD stages was also more frequent among ICI recipients, with stage IV CKD occurring in 8.8% of patients versus 8.3% in controls (HR 1.429; 95% CI, 1.377–1.484). Restricting the primary composite to its most specific components (N00, N01 and N04) yielded a hazard ratio of 1.797 (95% CI, 1.465–2.204), indicating that the association is not driven by the least specific codes (Supplementary Table S12).

### 3.5. Sensitivity Analyses

All sensitivity analyses consistently supported the primary findings. The on-treatment analysis, which required at least six administrations within the first six months in both cohorts and began the outcome window at day 180, comprised 34,762 matched pairs and yielded a hazard ratio of 1.623 (95% CI, 1.376–1.913). Exclusion of patients receiving nephrotoxic chemotherapy strengthened the association (HR 2.010; 95% CI, 1.843–2.191). Landmark analyses confirmed persistent associations after excluding events within 30 days (HR 1.504), 90 days (HR 1.480), and 180 days (HR 1.357), all of which remained statistically significant (Table 3, Supplementary Tables S13–S15). Negative control outcome analyses showed no significant associations for appendicitis (HR 0.934), suicide attempts (HR 0.854), or burns (HR 0.979), supporting the validity of our analytical approach. Conversely, both positive control outcomes were strongly associated with ICI exposure—thyroid dysfunction (HR 3.449; 95% CI, 3.348–3.554) and drug-induced colitis (HR 1.412; 95% CI, 1.372–1.454)—confirming that the analytic pipeline detects established immune-related adverse events and is not systematically biased towards the null (Supplementary Table S16).

Two further analyses addressed the specificity of the outcome and the possibility of differential detection. Within the on-treatment cohort, the magnitude of the association increased with outcome specificity: the hazard ratio was 1.623 for the six-code composite and 3.366 (95% CI, 1.855–6.107) for acute nephritic syndrome (N00 and N01), the most biologically specific component, whereas nephrotic syndrome alone (N04) did not significantly increase (HR 1.288; 95% CI, 0.917–1.810). A gradient in this direction is inconsistent with non-differential misclassification as the explanation for the association. With respect to detection, the two investigations by which glomerular disease is actually identified were performed at indistinguishable rates in the two cohorts: urine protein testing in 37.75% versus 38.01% (HR 0.993; 95% CI, 0.978–1.008) and renal biopsy in 0.56% versus 0.51% (HR 1.060; 95% CI, 0.936–1.165). Renal function testing was performed in virtually all patients in both cohorts (99.55% versus 99.97%) and therefore does not discriminate between them (Supplementary Table S17).

## 4. Discussion

This large, propensity score-matched cohort study provides real-world evidence that ICI therapy is associated with a higher hazard of coded incident glomerular disease than conventional cancer treatments and characterizes both the magnitude and the time course of that association. To our knowledge, this represents the most comprehensive comparative analysis to date of ICI-associated glomerular disease risk, filling a critical knowledge gap in the emerging field of onco-nephrology.

Three features of the association are important for its clinical interpretation. First, the relative effect is substantial, but the absolute effect is modest: the estimated difference in the 5-year cumulative incidence was approximately 1.2 percentage points, of the order of 12 additional cases per 1000 patients treated. Because these absolute estimates were derived from the survival functions of the matched cohort rather than from patient-level competing-risk regression, they should be read as approximations rather than as precise estimates of absolute harm. Second, the excess hazard is concentrated in the first 500 days, and the interval point estimates approach unity beyond roughly 1000 days, which argues for intensified surveillance during the first 18 months rather than indefinitely. Third, the association does not appear to be explained by the competing risk of death: mortality was nearly identical in the two cohorts, and the approximate subdistribution hazard ratio of 1.48 was close to the cause-specific estimate.

Our findings carry several important clinical implications. The observed 2.5% incidence of glomerular disease among ICI recipients exceeds rates previously reported in case reports and small series [20], raising concern that glomerular involvement may be under-recognized in routine clinical practice. Heterogeneity by race and by cancer type was supported by formal testing, and the higher estimates in Asian patients (HR 2.195) and in patients with female reproductive cancers (HR 2.303) may identify populations in whom closer monitoring is warranted [21]. These signals are nevertheless exploratory and require replication before they are used to direct surveillance. By contrast, the numerically higher estimates among users of proton pump inhibitors, NSAIDs and renin–angiotensin system inhibitors were homogeneous with the overall estimate and provide no evidence of effect modification by concomitant medication [22].

The strongest association among the glomerular subtypes was that for acute nephritic syndrome (HR 2.037), which is consistent with immune-mediated glomerular inflammation as a central pathophysiologic mechanism and contrasts with the tubulointerstitial injury typically observed in conventional drug-induced nephropathies [23]. Acute nephritic syndrome was nevertheless the least frequent of the subtypes examined, with 196 events in the immune checkpoint inhibitor cohort, so this observation rests on a small number of events. This interpretation is reinforced by the specificity gradient in the on-treatment analysis, in which the association was strongest for the nephritic component and absent for nephrotic syndrome alone. The temporal risk profile, with highest incidence within the first 500 days, parallels the onset timeline of other severe irAEs [24]. In contrast to our original interpretation, the interval analysis indicates that the excess hazard does not persist indefinitely; beyond approximately 1000 days the two cohorts were indistinguishable, which suggests that surveillance can reasonably be concentrated in the earlier period.

Our findings both corroborate and expand upon prior observations in the field. The overall nephrotoxicity rate identified in our cohort aligns with recent meta-analyses reporting an AKI incidence ranging from 2% to 7% among patients receiving ICI therapy [25]. However, by focusing specifically on glomerular disease, our study reveals a distinct risk profile that differs from that of overall AKI. A systematic review by Kitchlu et al. documented 45 cases of ICI-associated glomerular disease, with pauci-immune glomerulonephritis and podocytopathies identified as the most prevalent subtypes [8]. Our population-level analysis is consistent with previously reported histopathological patterns and extends these observations by providing comparative risk estimates relative to conventional cancer treatments.

The observed variation in glomerular disease risk by ICI agent—with atezolizumab demonstrating the highest risk—has not been previously reported and may reflect differences in target specificity, pharmacodynamics, or underlying patient populations. The estimate for ipilimumab monotherapy was below unity but rested on very few events, with a confidence interval spanning a sixfold range; it should not be interpreted as evidence against the nephrotoxicity linked to CTLA-4 inhibition in prior reports [26]. Both agent-level findings are post hoc and require confirmation.

Two limitations deserve particular emphasis because they bear directly on the interpretation of the primary finding. The first is outcome misclassification. The primary outcome was defined by ICD-10-CM codes for clinically recognized diagnoses, and these codes cannot distinguish with certainty between biopsy-confirmed glomerular disease, clinically suspected disease, and coding entered for administrative purposes. Kidney biopsy results, urine microscopy, quantitative proteinuria and serological testing are not uniformly available in the network, so individual diagnoses could not be validated histopathologically. Our findings should therefore be read as an association between ICI exposure and coded incident glomerular disease rather than biopsy-confirmed disease. Two observations argue against non-differential misclassification as the explanation: the association strengthened as the outcome definition was restricted to more-specific codes, and it was absent for the least mechanistically plausible component.

The second is detection bias. Patients receiving ICIs may undergo more-frequent laboratory monitoring because of concern about immune-related adverse events, which would increase the likelihood that renal abnormalities are detected and coded. We addressed this directly by comparing monitoring intensity between cohorts. Urine protein testing and renal biopsy, the two investigations through which glomerular disease is actually identified, were performed at indistinguishable rates, and renal function testing was near-universal in both cohorts. Differential surveillance is therefore unlikely to account for the observed association, although it cannot be excluded entirely because the platform does not capture the clinical reasoning behind each test.

This study has several strengths, including large sample size, rigorous propensity score matching, multiple sensitivity analyses, formal assessment of the proportional hazards assumption, a competing-risk analysis, and both negative and positive control outcomes. However, several further limitations warrant consideration. Detailed information on cancer staging, ICI dosing regimens, and specific chemotherapy protocols was unavailable, potentially limiting granular subgroup analyses. Despite comprehensive propensity score matching, residual confounding due to unmeasured variables cannot be entirely excluded, although the E-value indicates that an unmeasured confounder would need to be associated with both exposure and outcome by a risk ratio of at least 2.35 to explain the association away. The median follow-up of approximately two years may be insufficient to capture all late-onset events. Although the requirement for repeated administrations was applied symmetrically to both cohorts and a day-180 landmark analysis was performed, this design feature cannot be entirely eliminated as a source of selection bias in a database that lacks protocol-level treatment information [12]. Finally, the cohort was drawn from the TriNetX U.S. Collaborative Network, so the findings are representative of United States practice and may not generalize to other healthcare systems.

## 5. Conclusions

ICI therapy is associated with a higher hazard of coded incident glomerular disease than conventional cancer treatments, with an estimated absolute difference in 5-year cumulative incidence of the order of 1.2 percentage points in supporting competing-risk analyses. The excess hazard is concentrated in the first 500 days after initiation, with interval point estimates approaching unity beyond approximately 1000 days, and the association does not appear to be explained by the competing risk of death or by differential renal monitoring. In exploratory analyses, estimates were higher in Asian patients, in patients with female reproductive cancers and with atezolizumab, although these findings require replication. These results support heightened clinical awareness and targeted renal surveillance during the first 18 months of ICI therapy rather than indefinite monitoring of all recipients.

## Figures and Tables

**Table 1:** Baseline characteristics of patients before and after propensity score matching.

Characteristic	ICI Before	Non-ICI Before	SMD	ICI After	Non-ICI After	SMD
*n*	72,133	528,566		71,354	71,354	
Demographics						
Age at index, mean ± SD, years	65.0 ± 13.1	61.7 ± 15.3	0.235	65.0 ± 13.1	64.6 ± 13.3	0.033
Male	36,684 (50.9)	222,746 (42.4)	0.169	36,193 (50.7)	35,988 (50.4)	0.006
Female	32,082 (44.5)	287,044 (54.7)	0.205	31,862 (44.7)	32,444 (45.5)	0.016
Unknown	3367 (4.7)	15,022 (2.9)	0.095	3299 (4.6)	2922 (4.1)	0.026
Race/ethnicity						
White	54,020 (74.9)	391,233 (74.5)	0.008	53,406 (74.8)	53,346 (74.8)	0.002
Black or African American	6221 (8.6)	47,919 (9.1)	0.018	6177 (8.7)	6291 (8.8)	0.006
Asian	3336 (4.6)	22,423 (4.3)	0.017	3318 (4.7)	3473 (4.9)	0.010
Unknown	5505 (7.6)	40,821 (7.8)	0.005	5432 (7.6)	5096 (7.1)	0.018
Comorbidities						
Chronic kidney disease	6869 (9.5)	27,336 (5.2)	0.166	6703 (9.4)	6087 (8.5)	0.030
Type 2 diabetes mellitus	13,311 (18.5)	76,886 (14.7)	0.102	13,131 (18.4)	12,821 (18.0)	0.011
Hypertensive diseases	35,432 (49.1)	200,269 (38.2)	0.222	34,921 (48.9)	34,025 (47.7)	0.025
Ischaemic heart diseases	13,962 (19.4)	56,083 (10.7)	0.244	13,687 (19.2)	12,719 (17.8)	0.035
Liver diseases	13,518 (18.7)	50,534 (9.6)	0.263	13,240 (18.6)	12,946 (18.1)	0.011
Systemic lupus erythematosus	196 (0.3)	2201 (0.4)	0.025	196 (0.3)	217 (0.3)	0.005
Other connective tissue disorders	543 (0.8)	5572 (1.1)	0.033	540 (0.8)	467 (0.7)	0.012
Cancer types						
Digestive (gastrointestinal)	13,817 (19.2)	91,372 (17.4)	0.045	13,677 (19.2)	14,844 (20.8)	0.041
Thoracic (lung and respiratory)	27,734 (38.4)	40,977 (7.8)	0.780	27,006 (37.8)	26,126 (36.6)	0.026
Skin (melanoma)	1296 (1.8)	4667 (0.9)	0.079	1236 (1.7)	1110 (1.6)	0.014
Breast	5988 (8.3)	88,004 (16.8)	0.258	5959 (8.4)	6217 (8.7)	0.013
Male reproductive (genitourinary)	3189 (4.4)	29,247 (5.6)	0.053	3136 (4.4)	2806 (3.9)	0.023
Female reproductive (gynaecologic)	5770 (8.0)	33,696 (6.4)	0.061	5726 (8.0)	6239 (8.7)	0.026
Genitourinary (kidney/bladder)	9757 (13.5)	21,161 (4.0)	0.340	9441 (13.2)	9669 (13.6)	0.009
Blood (haematologic)	4181 (5.8)	106,488 (20.3)	0.441	4164 (5.8)	3355 (4.7)	0.051
Head and neck	3791 (5.3)	13,815 (2.6)	0.135	3665 (5.1)	3641 (5.1)	0.002
Other	4948 (7.0)	15,546 (6.9)	0.003	4423 (6.4)	4390 (6.3)	0.002
Concomitant medications						
Proton pump inhibitors	24,198 (33.5)	126,723 (24.1)	0.209	23,840 (33.4)	23,503 (32.9)	0.010
ACE inhibitors	10,232 (14.2)	60,127 (11.5)	0.082	10,102 (14.2)	9630 (13.5)	0.019
Angiotensin receptor blockers	8335 (11.6)	47,420 (9.0)	0.083	8214 (11.5)	7690 (10.8)	0.023
NSAIDs	10,664 (14.8)	59,163 (11.3)	0.104	10,532 (14.8)	10,281 (14.4)	0.010
Glucocorticoids	50,409 (69.9)	255,209 (48.6)	0.443	49,692 (69.6)	48,602 (68.1)	0.033
Thiazide diuretics	8479 (11.8)	54,603 (10.4)	0.043	8397 (11.8)	8002 (11.2)	0.017
Aldosterone antagonists	2682 (3.7)	15,323 (2.9)	0.045	2659 (3.7)	2524 (3.5)	0.010
Loop diuretics	10,138 (14.1)	42,575 (8.1)	0.190	9956 (14.0)	9472 (13.3)	0.020
Laboratory values						
Serum creatinine, mean ± SD, mg/dL	0.9 ± 0.3	0.9 ± 0.4	0.117	0.9 ± 0.3	0.9 ± 0.3	0.060
eGFR by MDRD, mean ± SD, mL/min/1.73 m^2^	82.9 ± 29.9	83.7 ± 28.0	0.026	82.9 ± 29.9	83.8 ± 29.8	0.028

Data are presented as mean ± SD for continuous variables and n (%) for categorical variables. SMD < 0.1 indicates adequate balance between groups after matching. Propensity score matching was performed using 1:1 greedy nearest-neighbour matching without replacement. SMD, standardized mean difference.

**Table 2:** Time course and absolute magnitude of the association between ICI therapy and incident glomerular disease.

Panel (A): Non-overlapping interval analysis					
**Interval**	**ICI Cumulative Hazard Increment**		**Non-ICI Cumulative Hazard Increment**		**Interval Hazard Ratio**
Day 1–500	0.0245		0.0146		1.68
Day 501–1000	0.0139		0.0100		1.39
Day 1001–1500	0.0106		0.0100		1.05
Day 1501–2000	0.0100		0.0085		1.17
Day 2001–3000	0.0091		0.0128		0.72
Panel (B): Cumulative incidence with death as a competing event, and absolute risk					
**Time Since Index**	**ICI Cumulative Incidence**	**Non-ICI Cumulative Incidence**	**Risk Difference (per 1000)**	**Number Needed to Harm**	**RMST Difference (Days, 95% CI)**
1 year	1.73%	1.01%	7.2	139	−1.72 (−2.08 to −1.36)
2 years	2.63%	1.68%	9.5	105	−5.42 (−6.43 to −4.41)
3 years	3.34%	2.24%	11.0	91	−10.05 (−11.93 to −8.17)
5 years	4.47%	3.23%	12.5	80	−20.28 (−24.63 to −15.93)

Panel (**A**): Each interval counts only the hazard accrued within that interval among patients still under observation at its start; the intervals are non-overlapping and are not cumulative landmark analyses. Panel (**B**): Cumulative incidence was estimated by the Aalen–Johansen method with death as a competing event. RMST, restricted mean survival time free of glomerular disease. Further detail is given in Supplementary Tables S19 and S20.

**Table 3:** Landmark sensitivity analysis of incident glomerular disease after excluding early events.

Landmark	ICI Events, n (%)	Non-ICI Events, n (%)	Risk Ratio (95% CI)	Hazard Ratio (95% CI)	*p*	E-Value
Day 30 onwards	1577/69,339 (2.3%)	1633/69,637 (2.3%)	0.970 (0.906–1.039)	1.504 (1.401–1.616)	<0.001	2.37
Day 90 onwards	1309/69,071 (1.9%)	1473/69,477 (2.1%)	0.894 (0.830–0.962)	1.480 (1.370–1.598)	<0.001	2.32
Day 180 onwards	980/68,742 (1.4%)	1318/69,322 (1.9%)	0.750 (0.691–0.814)	1.357 (1.246–1.478)	<0.001	2.05

Landmark analyses excluded patients who experienced the outcome, died, or were lost to follow-up before each landmark time point (30, 90 and 180 days after treatment initiation) to reduce potential immortal time bias. Hazard ratios were estimated using Cox proportional hazards regression. As elsewhere, the risk ratio falls below 1 at later landmarks because it is a crude cumulative proportion that does not account for differences in time at risk, whereas the hazard ratio, which compares instantaneous rates among patients still at risk, remains elevated.

## Data Availability

Data are available through the TriNetX platform subject to institutional data use agreements.

## Supplementary Materials

The following supporting information can be downloaded at https://www.mdpi.com/article/10.3390/life16091576/s1: Document S1: STROBE statement checklist; Table S1: Study period, index date and analysis time window; Table S2: Cohort inclusion and exclusion criteria with diagnostic codes; Table S3: Exposure definitions; Table S4: Primary outcome definition; Table S5: Secondary renal outcome definitions; Table S6: Negative and positive control outcome definitions; Table S7: Covariates used for propensity score matching; Table S8: Laboratory and procedure codes used for the monitoring-intensity analysis; Table S9: Baseline characteristics of the on-treatment design cohort; Table S10: Baseline characteristics of the cohort excluding nephrotoxic chemotherapy; Table S11: Baseline characteristics of the cohort excluding subsequent nephrotoxic chemotherapy; Table S12: Detailed secondary renal outcomes, main analysis; Table S13: Outcomes of the on-treatment design cohort (day-180 landmark); Table S14: Outcomes of ICI versus non-ICI without nephrotoxic chemotherapy; Table S15: Outcomes of ICI without subsequent nephrotoxic chemotherapy; Table S16: Negative and positive control outcomes; Table S17: Monitoring-intensity analysis; Table S18: Tests of heterogeneity across exploratory subgroups; Table S19: Competing-risk analysis and restricted mean survival time; Table S20: Non-overlapping interval analysis of the primary outcome; Methods S1: Additional methodological details.

## Abbreviations

ACE, angiotensin-converting enzyme; AKI, acute kidney injury; ARB, angiotensin receptor blocker; CI, confidence interval; CKD, chronic kidney disease; CTLA-4, cytotoxic T-lymphocyte-associated protein 4; eGFR, estimated glomerular filtration rate; HR, hazard ratio; ICD-10-CM, International Classification of Diseases, Tenth Revision, Clinical Modification; ICI, immune checkpoint inhibitor; irAE, immune-related adverse event; MDRD, Modification of Diet in Renal Disease; NSAID, nonsteroidal anti-inflammatory drug; PD-1, programmed death-1; PD-L1, programmed death-ligand 1; RMST, restricted mean survival time; SD, standard deviation; SMD, standardized mean difference.

## References

[1] Postow M.A., Sidlow R., Hellmann M.D. (2018). Immune-Related Adverse Events Associated with Immune Checkpoint Blockade. N. Engl. J. Med..

[2] Haslam A., Gill J., Prasad V. (2020). Estimation of the Percentage of US Patients With Cancer Who Are Eligible for Immune Checkpoint Inhibitor Drugs. JAMA Netw. Open.

[3] Martins F., Sofiya L., Sykiotis G.P., Lamine F., Maillard M., Fraga M., Shabafrouz K., Ribi C., Cairoli A., Guex-Crosier Y. (2019). Adverse effects of immune-checkpoint inhibitors: Epidemiology, management and surveillance. Nat. Rev. Clin. Oncol..

[4] Perazella M.A., Shirali A.C. (2020). Immune checkpoint inhibitor nephrotoxicity: What do we know and what should we do?. Kidney Int..

[5] Cortazar F.B., Kibbelaar Z.A., Glezerman I.G., Abudayyeh A., Mamlouk O., Motwani S.S., Murakami N., Herrmann S.M., Manohar S., Shirali A.C. (2020). Clinical Features and Outcomes of Immune Checkpoint Inhibitor-Associated AKI: A Multicenter Study. J. Am. Soc. Nephrol..

[6] Mamlouk O., Selamet U., Machado S., Abdelrahim M., Glass W.F., Tchakarov A., Gaber L., Lahoti A., Workeneh B., Chen S. (2019). Nephrotoxicity of immune checkpoint inhibitors beyond tubulointerstitial nephritis: Single-center experience. J. Immunother. Cancer.

[7] Izzedine H., Mathian A., Champiat S., Picard C., Mateus C., Routier E., Varga A., Malka D., Leary A., Michels J. (2019). Renal toxicities associated with pembrolizumab. Clin. Kidney J..

[8] Kitchlu A., Jhaveri K.D., Wadhwani S., Deshpande P., Harel Z., Kishibe T., Henriksen K., Wanchoo R. (2021). A Systematic Review of Immune Checkpoint Inhibitor-Associated Glomerular Disease. Kidney Int. Rep..

[9] He X., Liu F., Jin Y., Fu H., Mao J. (2022). Glomerular diseases after immune checkpoint inhibitors use: What do we know so far?. Ren. Fail..

[10] Herrmann S.M., Abudayyeh A., Gupta S., Gudsoorkar P., Klomjit N., Motwani S.S., Karam S., Costa E Silva V.T., Khalid S.B., Anand S. (2025). Diagnosis and management of immune checkpoint inhibitor-associated nephrotoxicity: A position statement from the American Society of Onco-nephrology. Kidney Int..

[11] Hernán M.A., Robins J.M. (2016). Using Big Data to Emulate a Target Trial When a Randomized Trial Is Not Available. Am. J. Epidemiol..

[12] Suissa S. (2008). Immortal time bias in pharmacoepidemiology. Am. J. Epidemiol..

[13] Austin P.C. (2011). An Introduction to Propensity Score Methods for Reducing the Effects of Confounding in Observational Studies. Multivar. Behav. Res..

[14] Royston P., Parmar M.K.B. (2013). Restricted mean survival time: An alternative to the hazard ratio for the design and analysis of randomized trials with a time-to-event outcome. BMC Med. Res. Methodol..

[15] Austin P.C., Lee D.S., Fine J.P. (2016). Introduction to the Analysis of Survival Data in the Presence of Competing Risks. Circulation.

[16] VanderWeele T.J., Ding P. (2017). Sensitivity Analysis in Observational Research: Introducing the E-Value. Ann. Intern. Med..

[17] Wang R., Lagakos S.W., Ware J.H., Hunter D.J., Drazen J.M. (2007). Statistics in Medicine—Reporting of Subgroup Analyses in Clinical Trials. N. Engl. J. Med..

[18] Benjamini Y., Hochberg Y. (1995). Controlling the False Discovery Rate: A Practical and Powerful Approach to Multiple Testing. J. R. Stat. Soc. Ser. B Stat. Methodol..

[19] Lipsitch M., Tchetgen Tchetgen E., Cohen T. (2010). Negative controls: A tool for detecting confounding and bias in observational studies. Epidemiology.

[20] Manohar S., Ghamrawi R., Chengappa M., Goksu B.N.B., Kottschade L., Finnes H., Dronca R., Leventakos K., Herrmann J., Herrmann S.M. (2020). Acute Interstitial Nephritis and Checkpoint Inhibitor Therapy: Single Center Experience of Management and Drug Rechallenge. Kidney360.

[21] Qin Z., Liu K., Xu X., Li T., Ge Y., Wu B., Xing C., Mao H. (2022). Incidence, predictors and 6-month overall outcome of acute kidney injury in Chinese patients receiving PD-1 inhibitors. Future Oncol..

[22] Mohan A., Krisanapan P., Tangpanithandee S., Thongprayoon C., Kanduri S.R., Cheungpasitporn W., Herrmann S.M. (2024). Association of Proton Pump Inhibitor Use and Immune Checkpoint Inhibitor-Mediated Acute Kidney Injury: A Meta-Analysis and a Review of Related Outcomes. Am. J. Nephrol..

[23] Miao J., Sise M.E., Herrmann S.M. (2022). Immune checkpoint inhibitor related nephrotoxicity: Advances in clinicopathologic features, noninvasive approaches, and therapeutic strategy and rechallenge. Front. Nephrol..

[24] Zhou P., Gao Y., Kong Z., Wang J., Si S., Han W., Li J., Lv Z., Wang R. (2024). Immune checkpoint inhibitors and acute kidney injury. Front. Immunol..

[25] Chen J.J., Lee T.H., Kuo G., Yen C.L., Lee C.C., Chang C.H., Tu K.H., Chen Y.C., Fang J.T., Hung C.C. (2024). All-cause and immune checkpoint inhibitor-associated acute kidney injury in immune checkpoint inhibitor users: A meta-analysis of occurrence rate, risk factors and mortality. Clin. Kidney J..

[26] Meraz-Muñoz A., Amir E., Ng P., Avila-Casado C., Ragobar C., Chan C., Kim J., Wald R., Kitchlu A. (2020). Acute kidney injury associated with immune checkpoint inhibitor therapy: Incidence, risk factors and outcomes. J. Immunother. Cancer.

