# Supplementary material for: Immune Checkpoint Inhibitor Therapy and Risk of Glomerular Disease: A Large Propensity-Matched Cohort Study
Source: Life (Basel). 2026 Sep 21;16(9):1576. doi: 10.3390/life16091576 (PMC13608369; doi:10.3390/life16091576)
Supplement: Supplementary file 1 [file life-16-01576-s001.zip › life-4519820-supplementary.pdf]

Supplementary Materials

Immune Checkpoint Inhibitor Therapy and Risk of Glomerular Disease: A Large Propensity-Matched Cohort Study

Ping-Huang Tsai, Wei-Cheng Chang, Hong-Jie Jhou, Hsin-Yu Chen, Li-Ting Kao, Tina Yi-Jin Hsieh, Po-Huang Chen, Cho-Hao Lee

Corresponding author: Cho-Hao Lee, MD. Division of Hematology and Oncology, Department of Internal Medicine, Tri-Service General Hospital, National Defense Medical University, Taipei, Taiwan. drleechohao@gmail.com

TriNetX US Collaborative Network | Study period 1 January 2014 – 1 March 2025 | Index eligibility 1 January 2014 – 1 March 2024

Contents

Item	Title
Document S1	STROBE statement checklist
**Study design, definitions and code lists**	
Table S1	Study period, index date and analysis time window
Table S2	Cohort inclusion and exclusion criteria with diagnostic codes
Table S3	Exposure definitions: immune checkpoint inhibitors and comparator agents
Table S4	Primary outcome definition
Table S5	Secondary renal outcome definitions
Table S6	Negative and positive control outcome definitions
Table S7	Covariates used for propensity score matching
Table S8	Laboratory and procedure codes used for the monitoring-intensity analysis
**Baseline characteristics of the sensitivity-analysis cohorts**	
Table S9	Baseline characteristics — on-treatment design cohort
Table S10	Baseline characteristics — ICI vs non-ICI without nephrotoxic chemotherapy
Table S11	Baseline characteristics — ICI without subsequent nephrotoxic chemotherapy
**Results**	
Table S12	Detailed secondary renal outcomes, main analysis
Table S13	Outcomes of the on-treatment design cohort (day-180 landmark)
Table S14	Outcomes — ICI vs non-ICI without nephrotoxic chemotherapy
Table S15	Outcomes — ICI without subsequent nephrotoxic chemotherapy
Table S16	Negative and positive control outcomes
Table S17	Monitoring-intensity analysis
Table S18	Tests of heterogeneity across exploratory subgroups
Table S19	Competing-risk analysis and restricted mean survival time
Table S20	Non-overlapping interval analysis of the primary outcome
**Methods**	
Methods S1	Additional methodological details

Document S1. STROBE statement checklist

Item	Recommendation	Reported in	Comment
**Title and abstract**			
1a	Indicate the study design with a commonly used term in the title or the abstract	Title; Abstract	Identified as a propensity-matched cohort study in the title
1b	Provide in the abstract an informative and balanced summary of what was done and what was found	Abstract	Background, methods, results and conclusions provided; absolute as well as relative measures reported
**Introduction**			
2	Explain the scientific background and rationale	Introduction ¶1–3	Background on ICI-associated nephrotoxicity and the knowledge gap for glomerular disease
3	State specific objectives, including any prespecified hypotheses	Introduction ¶4	Three objectives: quantify risk, identify high-risk subgroups, characterise timing
**Methods**			
4	Present key elements of study design early in the paper	Methods ¶1; Table S1	Retrospective new-user active-comparator cohort within a target trial emulation framework
5	Describe the setting, locations and relevant dates	Methods ¶1; Table S1	TriNetX US Collaborative Network, 74 healthcare organisations; study period 1 January 2014 – 1 March 2025; index eligibility to 1 March 2024
6a	Give eligibility criteria and the sources and methods of selection of participants; describe methods of follow-up	Methods, Study Population; Tables S1–S2	Adults ≥18 years with malignancy; exclusions with codes; follow-up to outcome, death or end of study
6b	For matched studies, give matching criteria and the number of exposed and unexposed	Methods, PSM; Table 1; Table S7	1:1 greedy nearest-neighbour matching on 40 covariates; 71,354 pairs
7	Clearly define all outcomes, exposures, predictors, potential confounders and effect modifiers	Methods; Tables S3–S7	Exposure by RxNorm/ATC; primary outcome N00, N01, N02, N04, N05, N08; all covariates defined with codes
8	For each variable of interest, give sources of data and details of methods of assessment	Methods; Tables S3–S8	EHR data; ICD-10-CM, RxNorm, ATC, LOINC, CPT; eGFR by MDRD
9	Describe any efforts to address potential sources of bias	Methods; Tables S1, S13, S16, S17	Active comparator; symmetric exposure criteria; index-date constraint; day-180 landmark; negative and positive controls; monitoring-intensity analysis
10	Explain how the study size was arrived at	Figure 1; Methods	All eligible patients in the network; 71,354 matched pairs achieved
11	Explain how quantitative variables were handled	Methods, Covariates	Age and eGFR continuous; categorical groupings defined for exploratory subgroup analyses
12a	Describe all statistical methods, including those used to control for confounding	Methods, Statistical Analysis; Methods S1	Propensity score matching; Cox proportional hazards regression with robust standard errors; proportional hazards assumption assessed for every outcome
12b	Describe any methods used to examine subgroups and interactions	Methods; Table S18	Cochran Q heterogeneity tests per domain with Benjamini–Hochberg adjustment; post-hoc pairwise contrasts
12c	Explain how missing data were addressed	Methods; Table 1	Unknown category retained for sex and race; complete case for laboratory values
12d	Explain how loss to follow-up was addressed	Methods; Table S19	Censoring at death, loss to follow-up or end of study; death additionally modelled as a competing event
12e	Describe any sensitivity analyses	Methods; Tables S13–S15, S19, S20	On-treatment analysis with day-180 landmark; exclusion of nephrotoxic chemotherapy; competing-risk analysis; restricted mean survival time; non-overlapping interval analysis
**Results**			
13	Report numbers of individuals at each stage, reasons for non-participation, and consider a flow diagram	Figure 1; Results ¶1	115,192,013 screened → 600,699 eligible → 72,133 ICI / 528,566 non-ICI → 71,354 matched pairs
14	Give characteristics of study participants and numbers with missing data	Table 1; Tables S9–S11	Demographics, comorbidities, cancer types, medications and laboratory values for both cohorts, before and after matching
14c	Summarise follow-up time	Results, Primary Outcome	Median 764 days (IQR 1,111) ICI; 692 days (IQR 1,257) non-ICI
15	Report numbers of outcome events or summary measures over time	Results; Table 2; Figure 2; Table S20	Event counts by cohort; non-overlapping interval analysis; Kaplan–Meier and Aalen–Johansen curves
16a	Give unadjusted and confounder-adjusted estimates and their precision	Results; Figures 3–4	HR 1.492 (95% CI 1.392–1.599) for the primary outcome; all subgroup estimates with 95% CI

Item	Recommendation	Reported in	Comment
16b	Report category boundaries when continuous variables were categorised	Figure 3; Methods	Age 18–65 vs >65; all subgroup definitions given in Methods
16c	If relevant, translate estimates of relative risk into absolute risk	Results; Table S19	Cumulative incidence 4.47% vs 3.23% at 5 years; risk difference 12.5 per 1,000; number needed to harm ≈80
17	Report other analyses done	Results; Figure 3; Tables S13–S20	Subgroup and heterogeneity testing, sensitivity analyses, competing-risk analysis, RMST, control outcomes, monitoring intensity
**Discussion**			
18	Summarise key results with reference to study objectives	Discussion ¶1	Elevated hazard concentrated in the first 500 days; exploratory subgroup heterogeneity; absolute risk quantified
19	Discuss limitations, taking into account sources of potential bias or imprecision	Discussion, Limitations; Methods S1	Outcome misclassification, detection bias, residual confounding, unavailable cancer staging, residual selection from the repeated-administration requirement
20	Give a cautious overall interpretation	Discussion ¶2–5	Findings framed as an association with coded incident glomerular disease; compared with prior literature
21	Discuss the generalisability of the study results	Discussion, Strengths	US Collaborative Network; real-world population broader than trial populations, but not internationally representative
**Other information**			
22	Give the source of funding and the role of the funders	Declarations, Funding	TSGH-D-114074 to C.-H.L.; no funder role in study conduct

Reference: von Elm E, Altman DG, Egger M, Pocock SJ, Gøtzsche PC, Vandenbroucke JP. The Strengthening the Reporting of Observational Studies in Epidemiology (STROBE) statement: guidelines for reporting observational studies. Lancet 2007;370:1453–1457.

Table S1. Study period, index date and analysis time window

Parameter	Specification	Rationale
Data source	TriNetX US Collaborative Network; 74 healthcare organisations queried, 74 responded	Federated de-identified electronic health record network
Study period	1 January 2014 – 1 March 2025	Coincides with clinical adoption of immune checkpoint inhibitors following the 2014 approvals of pembrolizumab and nivolumab
Index date	First administration of the index therapy occurring within the index eligibility window	New-user design; the date constraint is applied to the index event definition itself, not only to the cohort-qualifying criteria
Index eligibility window	1 January 2014 – 1 March 2024, applied identically to both cohorts	Guarantees a minimum of 12 months of potential follow-up for every patient and prevents asymmetric exposure ascertainment between arms. This window was applied in the original analysis but was not stated in the Methods section of the original submission
Exposure requirement	≥2 administrations of the index therapy, applied symmetrically to both cohorts	Confirms exposure and reduces misclassification from single coded records that may represent prescriptions not administered
Analysis time window	Day 1 after the index date until the outcome, death, loss to follow-up or the end of the study period	Curves are truncated at day 3,000 for display, beyond which few patients remain at risk
On-treatment sensitivity analysis	≥6 administrations within the first 6 months, applied symmetrically to both cohorts; outcome window begins at day 180	The day-180 landmark removes person-time and events occurring before exposure status can be ascertained
Censoring	Death, loss to follow-up, or end of the study period, whichever occurred first	Death is additionally modelled as a competing event (Table S19)

The index eligibility window was applied in the original analysis but was not described in the Methods section of the original submission; it is specified here in full. The date constraint applies to the index event definition itself and not only to the cohort-qualifying criteria, so that a patient cannot qualify for a cohort on the basis of treatment administered after the assigned time origin. The window and the requirement for repeated administrations are applied identically to the immune checkpoint inhibitor and comparator cohorts, so neither cohort has a longer opportunity to accrue qualifying exposure. Cohort sizes are therefore unchanged from the original submission.

Table S2. Cohort inclusion and exclusion criteria with diagnostic codes

Inclusion

Criterion	Code system	Codes
Age ≥18 years at index	Demographics	—
Confirmed malignant neoplasm	ICD-10-CM	C00–C14, C15–C26, C30–C39, C40–C41, C43–C44, C45–C49, C50, C51–C58, C60–C63, C64–C68, C69–C72, C73–C75, C81–C96

Malignancy categories: lip, oral cavity and pharynx (C00–C14); digestive organs (C15–C26); respiratory and intrathoracic organs (C30–C39); bone and articular cartilage (C40–C41); melanoma and other skin (C43–C44); mesothelial and soft tissue (C45–C49); breast (C50); female genital organs (C51–C58); male genital organs (C60–C63); urinary tract (C64–C68); eye, brain and central nervous system (C69–C72); thyroid and other endocrine glands (C73–C75); lymphoid and haematopoietic tissue (C81–C96).

Exclusion

Criterion	Code system	Codes	Timing
Pre-existing glomerular disease or end-stage renal disease	ICD-10-CM	N00, N01, N02, N03, N04, N05, N06, N07, N08, N18.6, Z99.2	Any instance on or before the first malignancy diagnosis
Acute kidney injury	ICD-10-CM	N17	Within 3 months on or before the index date
Severely reduced kidney function	Laboratory	eGFR (MDRD) ≤15 mL/min/1.73 m²	Within 3 months on or before the index date
Prior antineoplastic therapy before cancer diagnosis	RxNorm / ATC	Any index-cohort agent (Table S3)	First instance on or before the first malignancy diagnosis
Receipt of both ICI and non-ICI therapy during the initial treatment period	RxNorm / ATC	Cross-cohort exclusion	Initial treatment period

Exclusion of pre-existing glomerular disease was broadened relative to the original submission to include N03 (chronic nephritic syndrome), N06 (isolated proteinuria with specified morphological lesion), N07 (hereditary nephropathy), N18.6 (end-stage renal disease) and Z99.2 (dependence on renal dialysis), so that the analysis captures incident rather than prevalent disease. In addition, every outcome analysis excludes patients with that outcome recorded before the start of the time window; the numbers excluded are reported with each result.

Table S3. Exposure definitions

Immune checkpoint inhibitor cohort

Agent	Target	RxNorm CUI
Pembrolizumab	PD-1	1547545
Nivolumab	PD-1	1597876
Cemiplimab	PD-1	2058826
Atezolizumab	PD-L1	1792776
Durvalumab	PD-L1	1919503
Avelumab	PD-L1	1875534
Ipilimumab	CTLA-4	1094833
Relatlimab	LAG-3	2596773
Class-level term	PD-1 / PD-L1 inhibitors	ATC L01FF

Comparator cohort

ATC class	Description
L01A	Alkylating agents
L01B	Antimetabolites
L01C	Plant alkaloids and other natural products
L01D	Cytotoxic antibiotics and related substances
L01E	Protein kinase inhibitors
L01F	Monoclonal antibodies and antibody–drug conjugates
L01X	Other antineoplastic agents

Patients who received any immune checkpoint inhibitor listed above were excluded from the comparator cohort. L01FF (PD-1/PD-L1 inhibitors) is a subclass of L01F and was excluded from the comparator definition. Because L01E and L01F include vascular endothelial growth factor pathway inhibitors, which are themselves recognised causes of proteinuria and thrombotic microangiopathy, comparator-restricted analyses are reported separately (Tables S14 and S15).

Table S4. Primary outcome definition

ICD-10-CM	Description
N00	Acute nephritic syndrome
N01	Rapidly progressive nephritic syndrome
N02	Recurrent and persistent haematuria
N04	Nephrotic syndrome
N05	Unspecified nephritic syndrome
N08	Glomerular disorders in diseases classified elsewhere

The primary outcome comprises these six codes only. R80 (proteinuria) is not included: it is a sign rather than a glomerular disease diagnosis, it is separately analysed as a secondary outcome, and its inclusion would duplicate that component. N03, N06 and N07 are exclusion criteria rather than outcome components and are not counted as events. Patients with any primary-outcome code recorded before the start of the time window were excluded from the outcome analysis.

Table S5. Secondary renal outcome definitions

Outcome	Codes	Notes
Acute nephritic syndrome	N00, N01	Most biologically specific subset of the primary composite; N02, N03, N05 and N08 excluded
Nephrotic syndrome	N04	R80 excluded
Nephrotic syndrome, broad	N04, N05	R80 excluded
High-specificity glomerular disease	N00, N01, N04	Removes the least specific components of the primary composite
Tubulo-interstitial nephritis	N12, N14.1, N14.2	N10 (acute pyelonephritis) excluded as an infectious rather than drug-induced entity
Acute kidney injury	N17	—
Chronic kidney disease	N18.1, N18.2, N18.3, N18.4, N18.5	Parent code N18 not used, so that N18.6 and N18.9 are not counted

Outcome	Codes	Notes
CKD stage V / end-stage renal disease	N18.5, N18.6, Z99.2, or eGFR (MDRD) ≤15 mL/min/1.73 m²	—
Haematuria	R31	N02 excluded to avoid overlap with the primary outcome
Proteinuria	R80	Reported separately; not part of the primary outcome
Fluid, electrolyte and acid–base imbalance	E87	E83 excluded

Chronic kidney disease stages II, III and IV were additionally defined in earlier analyses by estimated glomerular filtration rate thresholds combined with diagnostic codes. Because an estimated glomerular filtration rate between 60 and 89 mL/min/1.73 m² is near-universal in this population, these laboratory-based stage definitions measure the distribution of kidney function rather than incident chronic kidney disease, and stages are therefore defined using diagnostic codes only.

Table S6. Negative and positive control outcome definitions

Negative controls

Outcome	Codes	Baseline exclusion
Appendicitis	K35, K36, K37	Prior appendicitis or appendicectomy
Suicide attempt	T14.91	—
Burn	T20–T25, T30–T32	—

Positive controls

Outcome	Codes	Baseline exclusion	Supporting measure
Thyroid dysfunction	E03.8, E03.9, E06.3, E06.9, E05	E00–E07 (any thyroid disorder)	New initiation of levothyroxine (RxNorm 10582)
Drug-induced colitis	K52.1, K52.89, K52.9	K50 (Crohn disease), K51 (ulcerative colitis)	—

F48 (other nonpsychotic mental disorders) was removed from the suicide-attempt definition used in the original submission; it denotes a broad category of nonpsychotic mental disorders and is not a marker of suicide attempt. Immune-related thyroid dysfunction and colitis are among the best established immune-related adverse events and are used to demonstrate that the analytic pipeline detects true immune-related events. Pneumonitis was not used as a positive control because respiratory and intrathoracic malignancies constitute a large proportion of the exposed cohort, so that J84 codes would be confounded by disease progression and radiation pneumonitis. Negative control outcomes were selected to have no plausible causal relationship with immune checkpoint inhibitor therapy.

Table S7. Covariates used for propensity score matching

Domain	Covariates	Codes
Demographics	Age at index; sex (male, female, unknown); race (White, Black or African American, Asian, unknown)	—
Comorbidities	Chronic kidney disease; type 2 diabetes mellitus; hypertensive disease; ischaemic heart disease; liver disease; systemic lupus erythematosus; other connective tissue disease	N18; E11; I10–I15; I20–I25; K70–K77; M32; M35
Malignancy type	Thirteen mutually exclusive site categories	C00–C14; C15–C26; C30–C39; C40–C41; C43–C44; C45–C49; C50; C51–C58; C60–C63; C64–C68; C69–C72; C73–C75; C81–C96
Concomitant medications	Proton pump inhibitors; angiotensin-converting enzyme inhibitors; angiotensin II receptor blockers; non-steroidal anti-inflammatory drugs; glucocorticoids; thiazide diuretics; aldosterone antagonists and potassium-sparing diuretics; loop diuretics	RxNorm / ATC class terms
Laboratory values	Serum creatinine; estimated glomerular filtration rate (MDRD)	LOINC 2160-0; TNX:8001

Forty covariates were entered into a multivariable logistic regression model. Matching was 1:1 greedy nearest-neighbour without replacement, with a caliper of 0.2 times the standard deviation of the logit of the propensity score. Balance was assessed by standardised mean difference, with values below 0.10 considered adequate; all covariates met this criterion after matching.

Variables assessed but not included in the propensity score model

Variable	Codes	Reason for assessment
Secondary (metastatic) malignant neoplasm	C77, C78, C79	Proxy for disease stage, which is incompletely captured in the network; imbalance would affect both mortality and monitoring intensity
Calendar year of index date	—	Uptake of immune checkpoint inhibitors increased over the study period, which may produce differential administrative censoring
Baseline proteinuria	R80; urine protein/creatinine and albumin/creatinine ratios	Plausibly the strongest single predictor of the primary outcome
Viral hepatitis	B18, B19	Established cause of secondary glomerular disease; the model contains liver disease but does not separate viral aetiology

Post-matching prevalence and standardised mean differences for these variables are reported to demonstrate balance on characteristics that were not matched upon.

Table S8. Laboratory and procedure codes used for the monitoring-intensity analysis

Measurement	LOINC	Alternative CPT
Serum creatinine	2160-0, 38483-4	82565
Estimated glomerular filtration rate (MDRD)	TNX:8001	—
Urinalysis	24357-6	81001, 81002, 81003

Measurement	LOINC	Alternative CPT
Urine protein, dipstick	5804-0	84156
Urine protein/creatinine ratio	2889-4, 14958-3	—
Urine albumin/creatinine ratio	9318-7, 14959-1	—
Renal biopsy, percutaneous	—	50200
Renal biopsy by surgical exposure of kidney	—	50205

Because the platform returns only the most recent value within a query interval, testing was quantified as the proportion of patients with at least one recorded value during follow-up and, for procedures, by counting procedure codes.

Table S9. Baseline characteristics — on-treatment design cohort

Characteristic	Before propensity score matching			After propensity score matching		
	ICI	Non-ICI	SMD	ICI	Non-ICI	SMD
n	35,638	243,754		34,762	34,762	
**Demographics**						
Age, mean ± SD, years	65.0 ± 12.9	59.4 ± 16.3	0.382	64.8 ± 13.0	64.6 ± 13.1	0.018
Male	18,838 (52.9%)	104,942 (43.0%)	0.190	18,232 (52.4%)	17,936 (51.6%)	0.017
Female	16,781 (47.1%)	136,707 (56.1%)	0.190	16,513 (47.5%)	16,802 (48.3%)	0.017
**Race / ethnicity**						
White	27,845 (78.1%)	178,163 (73.1%)	0.104	27,110 (78.0%)	26,916 (77.4%)	0.013
Black or African American	3,229 (9.1%)	25,696 (10.5%)	0.053	3,171 (9.1%)	3,202 (9.2%)	0.003
Asian	1,956 (5.5%)	13,633 (5.6%)	0.007	1,922 (5.5%)	2,054 (5.9%)	0.016
Unknown	2,608 (7.3%)	26,262 (10.8%)	0.122	2,559 (7.4%)	2,590 (7.5%)	0.004
**Comorbidities**						
Chronic kidney disease	3,217 (9.0%)	10,882 (4.5%)	0.181	3,033 (8.7%)	2,875 (8.3%)	0.016
Type 2 diabetes mellitus	6,504 (18.3%)	35,036 (14.4%)	0.102	6,301 (18.1%)	6,343 (18.2%)	0.003
Hypertensive diseases	17,390 (48.8%)	87,978 (36.1%)	0.253	16,831 (48.4%)	16,591 (47.7%)	0.014
Ischaemic heart diseases	6,819 (19.1%)	23,584 (9.7%)	0.269	6,457 (18.6%)	6,155 (17.7%)	0.023
Liver diseases	6,259 (17.6%)	24,940 (10.2%)	0.210	5,897 (17.0%)	5,961 (17.1%)	0.005
Systemic lupus erythematosus	97 (0.3%)	895 (0.4%)	0.017	97 (0.3%)	106 (0.3%)	0.005
**Cancer types**						
Head and neck	1,698 (4.8%)	6,540 (2.7%)	0.109	1,631 (4.7%)	1,796 (5.2%)	0.022
Digestive (gastrointestinal)	6,264 (17.6%)	48,766 (20.0%)	0.066	6,179 (17.8%)	6,456 (18.6%)	0.021
Thoracic (lung and respiratory)	14,021 (39.3%)	18,881 (7.7%)	0.800	13,278 (38.2%)	12,970 (37.3%)	0.018
Skin (melanoma)	7,001 (19.6%)	11,951 (4.9%)	0.459	6,613 (19.0%)	6,613 (19.0%)	<0.001
Breast	3,112 (8.7%)	43,587 (17.9%)	0.276	3,095 (8.9%)	3,317 (9.5%)	0.022
Genitourinary	4,983 (14.0%)	9,749 (4.0%)	0.353	4,778 (13.7%)	4,927 (14.2%)	0.012
Blood (haematologic)	1,967 (5.5%)	66,902 (27.4%)	0.624	1,956 (5.6%)	1,610 (4.6%)	0.045
**Concomitant medications**						
Proton pump inhibitors	11,166 (31.3%)	59,631 (24.5%)	0.149	10,792 (31.0%)	10,742 (30.9%)	0.003
ACE inhibitors	4,914 (13.8%)	26,543 (10.9%)	0.085	4,772 (13.7%)	4,627 (13.3%)	0.012
Angiotensin receptor blockers	4,142 (11.6%)	19,595 (8.0%)	0.118	3,995 (11.5%)	3,869 (11.1%)	0.011
NSAIDs	5,119 (14.4%)	30,638 (12.6%)	0.049	4,982 (14.3%)	4,894 (14.1%)	0.007
Glucocorticoids	24,389 (68.4%)	122,091 (50.1%)	0.371	23,589 (67.9%)	23,298 (67.0%)	0.018
Thiazide diuretics	4,193 (11.8%)	24,335 (10.0%)	0.054	4,088 (11.8%)	3,944 (11.3%)	0.013
Loop diuretics	4,586 (12.9%)	18,562 (7.6%)	0.172	4,373 (12.6%)	4,254 (12.2%)	0.010
**Laboratory values**						
Serum creatinine, mg/dL	0.9 ± 0.3	0.9 ± 0.3	0.191	0.9 ± 0.3	0.9 ± 0.3	0.070
eGFR (MDRD), mL/min/1.73 m²	81.9 ± 28.2	85.7 ± 28.1	0.136	81.9 ± 28.2	83.4 ± 28.7	0.051

The on-treatment design required ≥6 administrations of the index therapy within the first 6 months, applied symmetrically to both cohorts. A standardised mean difference (SMD) below 0.10 indicates adequate balance. Outcome analyses in this cohort begin at day 180 (Table S13).

Table S10. Baseline characteristics — ICI vs non-ICI without nephrotoxic chemotherapy

Characteristic	Before propensity score matching			After propensity score matching		
	ICI	Non-ICI	SMD	ICI	Non-ICI	SMD
n	70,659	223,754		48,551	48,551	
**Demographics**						
Age, mean ± SD, years	65.1 ± 13.1	64.7 ± 14.4	0.031	64.6 ± 13.9	64.3 ± 14.2	0.024
Male	35,830 (50.7%)	100,456 (46.1%)	0.093	24,185 (49.8%)	23,923 (49.3%)	0.011
Female	31,462 (44.5%)	112,641 (51.6%)	0.143	22,493 (46.3%)	22,813 (47.0%)	0.013
**Race / ethnicity**						
White	52,622 (74.5%)	172,394 (79.0%)	0.108	36,544 (75.3%)	36,555 (75.3%)	0.001
Black or African American	6,150 (8.7%)	16,629 (7.6%)	0.039	4,084 (8.4%)	4,197 (8.6%)	0.008
Asian	3,328 (4.7%)	6,275 (2.9%)	0.096	2,182 (4.5%)	2,171 (4.5%)	0.001
Unknown	5,493 (7.8%)	15,582 (7.1%)	0.024	3,575 (7.4%)	3,455 (7.1%)	0.010
**Comorbidities**						
Chronic kidney disease	6,896 (9.8%)	12,774 (5.9%)	0.146	4,367 (9.0%)	4,204 (8.7%)	0.012
Type 2 diabetes mellitus	13,247 (18.7%)	32,645 (15.0%)	0.101	8,890 (18.3%)	8,912 (18.4%)	0.001
Hypertensive diseases	35,126 (49.7%)	90,106 (41.3%)	0.169	22,962 (47.3%)	22,546 (46.4%)	0.017
Ischaemic heart diseases	13,980 (19.8%)	25,953 (11.9%)	0.217	8,178 (16.8%)	7,971 (16.4%)	0.011
Liver diseases	13,374 (18.9%)	17,066 (7.8%)	0.331	8,557 (17.6%)	8,700 (17.9%)	0.008
Systemic lupus erythematosus	192 (0.3%)	704 (0.3%)	0.009	130 (0.3%)	153 (0.3%)	0.009
**Cancer types**						
Head and neck	3,695 (5.2%)	2,343 (1.1%)	0.240	1,823 (3.8%)	1,655 (3.4%)	0.019
Digestive (gastrointestinal)	13,537 (19.2%)	32,464 (14.9%)	0.114	11,129 (22.9%)	12,454 (25.7%)	0.064
Thoracic (lung and respiratory)	27,737 (39.3%)	8,102 (3.7%)	0.960	9,684 (19.9%)	8,049 (16.6%)	0.087
Skin (melanoma)	13,399 (19.0%)	32,792 (15.0%)	0.105	10,284 (21.2%)	10,816 (22.3%)	0.027
Breast	5,974 (8.5%)	24,427 (11.2%)	0.092	4,969 (10.2%)	5,792 (11.9%)	0.054
Genitourinary	9,462 (13.4%)	10,702 (4.9%)	0.298	6,928 (14.3%)	7,722 (15.9%)	0.046
Blood (haematologic)	4,150 (5.9%)	26,583 (12.2%)	0.222	3,026 (6.2%)	2,591 (5.3%)	0.038
**Concomitant medications**						
Proton pump inhibitors	24,110 (34.1%)	50,547 (23.2%)	0.244	15,135 (31.2%)	15,033 (31.0%)	0.005
ACE inhibitors	10,295 (14.6%)	27,107 (12.4%)	0.063	6,690 (13.8%)	6,595 (13.6%)	0.006
Angiotensin receptor blockers	8,376 (11.9%)	22,618 (10.4%)	0.047	5,633 (11.6%)	5,442 (11.2%)	0.012
NSAIDs	10,699 (15.1%)	18,515 (8.5%)	0.207	6,695 (13.8%)	6,664 (13.7%)	0.002
Glucocorticoids	49,764 (70.4%)	92,802 (42.5%)	0.586	30,461 (62.7%)	29,959 (61.7%)	0.021
Thiazide diuretics	8,510 (12.0%)	25,267 (11.6%)	0.014	5,680 (11.7%)	5,387 (11.1%)	0.019
Loop diuretics	2,714 (3.8%)	7,328 (3.4%)	0.026	1,950 (4.0%)	1,905 (3.9%)	0.005
**Laboratory values**						
Serum creatinine, mg/dL	0.9 ± 0.3	0.9 ± 0.4	0.050	0.9 ± 0.4	0.9 ± 0.3	0.066
eGFR (MDRD), mL/min/1.73 m²	82.8 ± 29.8	80.8 ± 26.6	0.069	82.0 ± 29.5	82.4 ± 28.9	0.014

Excludes patients who received nephrotoxic chemotherapy in either cohort. SMD <0.10 indicates adequate balance.

Table S11. Baseline characteristics — ICI without subsequent nephrotoxic chemotherapy vs non-ICI without nephrotoxic chemotherapy

Characteristic	Before propensity score matching			After propensity score matching		
	ICI	Non-ICI	SMD	ICI	Non-ICI	SMD
n	33,475	223,754		31,982	31,982	
**Demographics**						
Age, mean ± SD, years	66.6 ± 13.9	64.7 ± 14.4	0.137	66.6 ± 14.0	66.4 ± 13.7	0.011
Male	19,877 (59.4%)	100,456 (46.1%)	0.269	18,842 (58.9%)	18,815 (58.8%)	0.002
Female	12,337 (36.9%)	112,641 (51.6%)	0.301	11,960 (37.4%)	12,060 (37.7%)	0.006
Unknown	1,261 (3.8%)	5,035 (2.3%)	0.085	1,180 (3.7%)	1,107 (3.5%)	0.012
**Race / ethnicity**						
White	25,986 (77.6%)	172,394 (79.0%)	0.034	24,818 (77.6%)	24,932 (78.0%)	0.009
Black or African American	2,430 (7.3%)	16,629 (7.6%)	0.014	2,356 (7.4%)	2,331 (7.3%)	0.003
Asian	1,356 (4.1%)	6,275 (2.9%)	0.064	1,283 (4.0%)	1,270 (4.0%)	0.002
Unknown	2,253 (6.7%)	15,582 (7.1%)	0.016	2,153 (6.7%)	2,093 (6.5%)	0.008
**Comorbidities**						
Chronic kidney disease	4,804 (14.4%)	12,774 (5.9%)	0.285	4,356 (13.6%)	3,806 (11.9%)	0.052
Type 2 diabetes mellitus	6,914 (20.7%)	32,645 (15.0%)	0.149	6,489 (20.3%)	6,357 (19.9%)	0.010
Hypertensive diseases	17,028 (50.9%)	90,106 (41.3%)	0.193	16,066 (50.2%)	15,541 (48.6%)	0.033
Ischaemic heart diseases	6,702 (20.0%)	25,953 (11.9%)	0.223	6,216 (19.4%)	6,100 (19.1%)	0.009
Liver diseases	6,516 (19.5%)	17,066 (7.8%)	0.344	5,991 (18.7%)	5,811 (18.2%)	0.015
Systemic lupus erythematosus	63 (0.2%)	704 (0.3%)	0.027	60 (0.2%)	89 (0.3%)	0.019
**Cancer types**						
Head and neck	1,350 (4.0%)	2,343 (1.1%)	0.188	1,125 (3.5%)	1,008 (3.2%)	0.020
Digestive (gastrointestinal)	6,203 (18.5%)	32,464 (14.9%)	0.098	5,955 (18.6%)	6,651 (20.8%)	0.055
Thoracic (lung and respiratory)	7,411 (22.1%)	8,102 (3.7%)	0.571	6,463 (20.2%)	6,263 (19.6%)	0.016
Skin (melanoma)	11,250 (33.6%)	32,792 (15.0%)	0.443	10,521 (32.9%)	10,927 (34.2%)	0.027
Breast	1,280 (3.8%)	24,427 (11.2%)	0.283	1,259 (3.9%)	1,241 (3.9%)	0.003
Genitourinary	7,942 (23.7%)	10,702 (4.9%)	0.558	7,209 (22.5%)	7,084 (22.1%)	0.009
Blood (haematologic)	1,832 (5.5%)	26,583 (12.2%)	0.238	1,755 (5.5%)	1,649 (5.2%)	0.015
**Concomitant medications**						
Proton pump inhibitors	10,139 (30.3%)	50,547 (23.2%)	0.161	9,522 (29.8%)	9,289 (29.0%)	0.016
ACE inhibitors	5,206 (15.6%)	27,107 (12.4%)	0.090	4,906 (15.3%)	4,686 (14.7%)	0.019
Angiotensin receptor blockers	4,305 (12.9%)	22,618 (10.4%)	0.078	4,065 (12.7%)	3,801 (11.9%)	0.025
NSAIDs	4,139 (12.4%)	18,515 (8.5%)	0.127	3,865 (12.1%)	3,792 (11.9%)	0.007
Glucocorticoids	18,528 (55.3%)	92,802 (42.5%)	0.258	17,449 (54.6%)	17,162 (53.7%)	0.018
Thiazide diuretics	4,049 (12.1%)	25,267 (11.6%)	0.016	3,840 (12.0%)	3,640 (11.4%)	0.019
Loop diuretics	1,709 (5.1%)	7,328 (3.4%)	0.087	1,613 (5.0%)	1,548 (4.8%)	0.009
**Laboratory values**						
Serum creatinine, mg/dL	1.0 ± 0.4	0.9 ± 0.4	0.392	1.0 ± 0.4	1.0 ± 0.4	0.041
eGFR (MDRD), mL/min/1.73 m²	78.5 ± 30.2	80.8 ± 26.6	0.359	78.8 ± 30.2	79.8 ± 29.5	0.038

Excludes immune checkpoint inhibitor recipients who received subsequent nephrotoxic chemotherapy and comparator patients who received nephrotoxic chemotherapy. SMD <0.10 indicates adequate balance.

Table S12. Detailed secondary renal outcomes, main analysis

Outcome	ICI events, n (%)	Non-ICI events, n (%)	Risk ratio (95% CI)	Hazard ratio (95% CI)
**Glomerular disease, by specificity**				
High-specificity glomerular disease (N00, N01, N04)	226 (0.32%)	169 (0.24%)	1.337 (1.096–1.632)	1.797 (1.465–2.204)
Acute nephritic syndrome	196 (0.3%)	135 (0.2%)	1.452 (1.166–1.807)	2.037 (1.627–2.551)
Nephrotic syndrome	1,557 (2.2%)	1,566 (2.2%)	0.998 (0.931–1.070)	1.523 (1.417–1.638)
**Tubulo-interstitial disease**				
Tubulo-interstitial nephritis	1,379 (2.0%)	1,191 (1.7%)	1.163 (1.077–1.256)	1.634 (1.509–1.769)
**Other renal outcomes**				
Acute kidney injury	11,487 (17.6%)	10,444 (15.5%)	1.137 (1.109–1.164)	1.507 (1.467–1.549)
Chronic kidney disease	5,988 (9.5%)	6,090 (9.5%)	0.999 (0.966–1.034)	1.490 (1.437–1.546)
CKD stage III	12,181 (27.5%)	13,729 (28.7%)	0.959 (0.939–0.979)	1.200 (1.171–1.230)
CKD stage IV	5,947 (8.8%)	5,662 (8.3%)	1.064 (1.028–1.102)	1.429 (1.377–1.484)
CKD stage V / ESRD	2,747 (3.9%)	2,729 (3.9%)	1.014 (0.963–1.068)	1.436 (1.360–1.516)
Haematuria	3,202 (5.1%)	4,105 (6.6%)	0.776 (0.742–0.812)	1.115 (1.064–1.169)
Proteinuria	1,329 (1.9%)	1,460 (2.1%)	0.914 (0.849–0.984)	1.418 (1.313–1.531)
Fluid, electrolyte and acid–base imbalance	17,398 (33.6%)	16,662 (29.4%)	1.143 (1.123–1.164)	1.455 (1.424–1.487)

All analyses were performed after 1:1 propensity score matching (71,354 pairs). Risk ratios above 1.0 indicate a higher cumulative proportion in the immune checkpoint inhibitor cohort; hazard ratios were estimated by Cox proportional hazards regression. For every outcome, patients with that outcome recorded before the start of the time window were excluded from the analysis of that outcome, so the denominator of each percentage is the number of patients at risk for that outcome rather than the full matched cohort; the denominators therefore differ between outcomes and between cohorts. The risk ratio is a crude cumulative proportion that does not account for differences in time at risk, and the hazard ratio should be treated as the primary effect estimate.

Table S13. Outcomes of the on-treatment design cohort (day-180 landmark)

Outcome	ICI events / N (%)	Non-ICI events / N (%)	Risk ratio (95% CI)	Hazard ratio (95% CI)	PH test P
**Primary outcome**					
Glomerular disease	346 / 34,762 (1.0%)	260 / 34,762 (0.7%)	1.331 (1.134–1.562)	1.623 (1.376–1.913)	0.003
**Secondary renal outcomes**					
Acute nephritic syndrome (N00, N01)	43 / 34,762 (0.12%)	16 / 34,762 (0.05%)	2.688 (1.514–4.770)	3.366 (1.855–6.107)	0.109
Nephrotic syndrome (N04)	71 / 34,762 (0.20%)	67 / 34,762 (0.19%)	1.060 (0.759–1.479)	1.288 (0.917–1.810)	0.241
Tubulo-interstitial nephritis	786 / 34,762 (2.3%)	793 / 34,762 (2.3%)	0.991 (0.899–1.093)	1.216 (1.100–1.345)	0.010
Acute kidney injury	6,349 / 34,762 (18.3%)	5,724 / 34,762 (16.5%)	1.109 (1.074–1.146)	1.396 (1.346–1.448)	<0.001
Chronic kidney disease	3,488 / 34,762 (10.0%)	3,725 / 34,762 (10.7%)	0.936 (0.896–0.978)	1.187 (1.132–1.244)	<0.001
Fluid, electrolyte and acid–base imbalance	8,557 / 34,762 (24.6%)	7,370 / 34,762 (21.2%)	1.161 (1.130–1.193)	1.531 (1.483–1.581)	<0.001

The on-treatment design required ≥6 administrations of the index therapy within the first 6 months, applied symmetrically to both cohorts, and the outcome window begins at day 180. This landmark removes person-time and events occurring before exposure status can be ascertained and is the analysis that addresses potential immortal time bias arising from the repeated-administration requirement. The cohort comprises 34,762 matched pairs, and the denominator for every outcome is the full matched cohort in each arm. Mean follow-up after matching was 1,170.0 ± 688.2 days (median 1,085) in the immune checkpoint inhibitor cohort and 1,446.2 ± 1,034.2 days (median 1,230) in the comparator cohort. All outcomes use the revised definitions in Tables S4 and S5. PH test P denotes the P value of the test of the proportional hazards assumption; $P < 0.05$ indicates violation, and for those outcomes the reported hazard ratio represents a time-averaged effect.

The magnitude of the association increases as outcome specificity increases: the hazard ratio is 1.623 for the six-code composite and 3.366 for acute nephritic syndrome (N00, N01), the most biologically specific component, whereas nephrotic syndrome alone (N04) is not significantly increased. This gradient is inconsistent with non-differential outcome misclassification or with ascertainment artefact as the explanation for the association.

Table S14. Outcomes — ICI vs non-ICI without nephrotoxic chemotherapy

Outcome	ICI events, n (%)	Non-ICI events, n (%)	Risk ratio (95% CI)	Hazard ratio (95% CI)
Glomerular disease	1,348 (2.8%)	1,108 (2.3%)	1.216 (1.126–1.314)	2.010 (1.843–2.191)
Acute nephritic syndrome	333 (0.7%)	234 (0.5%)	1.422 (1.204–1.680)	2.121 (1.785–2.519)
Tubulo-interstitial nephritis	968 (2.0%)	757 (1.6%)	1.281 (1.166–1.407)	1.979 (1.792–2.184)
Acute kidney injury	7,817 (16.1%)	5,693 (11.7%)	1.402 (1.358–1.447)	2.056 (1.985–2.130)
Fluid, electrolyte and acid–base imbalance	11,916 (24.5%)	8,340 (17.2%)	1.520 (1.484–1.557)	2.223 (2.160–2.289)

48,551 matched pairs. Excludes patients who received nephrotoxic chemotherapy in either cohort.

Table S15. Outcomes — ICI without subsequent nephrotoxic chemotherapy

Outcome	ICI events, n (%)	Non-ICI events, n (%)	Risk ratio (95% CI)	Hazard ratio (95% CI)
Glomerular disease	1,060 (3.3%)	877 (2.7%)	1.209 (1.108–1.318)	1.905 (1.711–2.120)
Acute nephritic syndrome	283 (0.9%)	186 (0.6%)	1.521 (1.265–1.828)	2.161 (1.789–2.610)
Tubulo-interstitial nephritis	630 (2.0%)	520 (1.6%)	1.215 (1.083–1.363)	1.822 (1.616–2.054)
Acute kidney injury	4,827 (15.1%)	3,989 (12.5%)	1.303 (1.253–1.354)	1.895 (1.815–1.979)
Fluid, electrolyte and acid–base imbalance	7,107 (22.2%)	5,487 (17.2%)	1.338 (1.298–1.380)	1.953 (1.884–2.025)

31,982 matched pairs. Excludes immune checkpoint inhibitor recipients who received subsequent nephrotoxic chemotherapy and all patients who received nephrotoxic chemotherapy.

Table S16. Negative and positive control outcomes

Control type	Outcome	ICI events / N	Non-ICI events / N	Hazard ratio (95% CI)	P
**Negative controls — no association expected**					
Negative	Appendicitis	192 / 71,354 (0.27%)	294 / 71,354 (0.41%)	0.934 (0.776–1.125)	0.474
Negative	Suicide attempt	99 / 71,354 (0.14%)	180 / 71,354 (0.25%)	0.854 (0.664–1.100)	0.221
Negative	Burn	285 / 71,354 (0.40%)	427 / 71,354 (0.60%)	0.979 (0.839–1.143)	0.789
**Positive controls — established immune-related adverse events**					
Positive	Thyroid dysfunction	15,659 / 71,354 (21.95%)	6,307 / 71,354 (8.84%)	3.449 (3.348–3.554)	<0.001
Positive	Drug-induced colitis	9,962 / 71,354 (13.96%)	8,615 / 71,354 (12.07%)	1.412 (1.372–1.454)	<0.001

Control outcomes are reported for the main analysis only, with all 71,354 matched patients per cohort retained. The absence of any association with three outcomes that have no plausible causal relationship to immune checkpoint inhibitor therapy argues against substantial residual confounding after matching. The strong association with thyroid dysfunction and drug-induced colitis, both well established immune-related adverse events, demonstrates that the analytic pipeline detects true immune-related events and is therefore not systematically biased towards the null.

Table S17. Monitoring-intensity analysis

Measure	ICI, n (%)	Non-ICI, n (%)	Risk ratio (95% CI)	Hazard ratio (95% CI)	Log-rank P
Any renal function testing	71,036 (99.55%)	71,331 (99.97%)	0.994 (0.994–0.995)	1.008 (1.006–1.011)	<0.001
Any urine protein testing	26,937 (37.75%)	27,123 (38.01%)	0.993 (0.980–1.006)	0.993 (0.978–1.008)	0.36
Renal biopsy	400 (0.56%)	367 (0.51%)	1.090 (0.946–1.255)	1.060 (0.936–1.165)	0.36

71,354 matched patients per cohort; all matched patients were retained for these measures. Differential surveillance is the principal alternative explanation for an apparent association between immune checkpoint inhibitor therapy and coded glomerular disease. Urine protein testing and renal biopsy, the two investigations by which glomerular disease is actually identified, were performed at indistinguishable rates in the two cohorts. Renal function testing was performed in virtually every patient in both cohorts, so this measure is at a ceiling and does not discriminate between them; it is reported to document that both cohorts were comprehensively monitored rather than as a test of differential surveillance. Taken together, these results do not support detection bias as the explanation for the observed association.

Table S18. Tests of heterogeneity across exploratory subgroups

Part A. Heterogeneity within each subgroup domain

Domain	Strata (k)	Cochran Q	df	P	I^2 (%)	BH-adjusted q
Cancer type	8	64.92	7	<0.001	89	<0.001
ICI agent	5	23.70	4	<0.001	83	<0.001
Diabetes mellitus	2	11.85	1	0.001	92	0.002
Ischaemic heart disease	2	10.51	1	0.001	90	0.003
Race	3	10.42	2	0.006	81	0.012
Chronic kidney disease	2	2.90	1	0.089	66	0.162
Liver disease	2	2.29	1	0.131	56	0.202
Sex	2	2.11	1	0.147	53	0.202
Hypertension	2	1.64	1	0.200	39	0.244
Regimen (single vs dual)	2	1.45	1	0.228	31	0.251
Age (18–65 vs >65)	2	0.00	1	1.000	0	1.000

Part B. Post-hoc pairwise contrasts

Domain	Contrast	Ratio of hazard ratios (95% CI)	P
Race	Asian vs White	1.63 (1.20–2.21)	0.002
Race	Asian vs Black	1.40 (0.97–2.03)	0.074
Race	Black vs White	1.16 (0.91–1.48)	0.227
Cancer type	Female reproductive vs haematologic	2.51 (1.82–3.47)	<0.001
Cancer type	Female reproductive vs skin	2.14 (1.68–2.73)	<0.001
Cancer type	Female reproductive vs lung and respiratory	1.73 (1.39–2.16)	<0.001
Cancer type	Female reproductive vs gastrointestinal	1.34 (1.07–1.68)	0.011
Cancer type	Female reproductive vs genitourinary	1.31 (1.06–1.64)	0.014
Cancer type	Female reproductive vs breast	1.29 (0.93–1.78)	0.128
ICI agent	Atezolizumab vs nivolumab	2.02 (1.42–2.86)	<0.001
ICI agent	Atezolizumab vs pembrolizumab	1.74 (1.28–2.37)	<0.001
ICI agent	Atezolizumab vs ipilimumab	4.00 (1.55–10.29)	0.004

Heterogeneity across the strata of each domain was tested by Cochran Q on the log hazard ratio scale, with weights equal to the inverse variance derived from the reported 95% confidence intervals (Figure 3). One test was performed per domain and P values were adjusted across the eleven domains by the Benjamini–Hochberg procedure; pairwise contrasts in Part B are post-hoc and are presented without further adjustment. I^2 denotes the proportion of between-stratum variation not attributable to sampling error.

Heterogeneity by race and by cancer type supports the higher estimates observed in Asian patients and in patients with female reproductive cancers. Heterogeneity by diabetes mellitus (hazard ratio 1.201 with diabetes vs 1.611 without) and by ischaemic heart disease (1.073 vs 1.478) indicates attenuation of the association in patients with a higher background burden of coded renal disease. The six concomitant medication subgroups were homogeneous ($Q = 1.54$, $df = 5$, $P = 0.909$, $I^2 = 0\%$) and each confidence interval included the overall estimate; these subgroups comprise medication users only, without a non-user reference stratum, and therefore do not constitute a test of effect modification. All subgroup analyses are exploratory: they were not prespecified in a statistical analysis plan, matching was not repeated within strata, and they should be interpreted as hypothesis-generating.

Table S19. Competing-risk analysis and restricted mean survival time

Part A. Cumulative incidence of glomerular disease with death as a competing event

Time since index	Kaplan–Meier cumulative probability		Aalen–Johansen cumulative incidence			All-cause mortality	
	ICI	Non-ICI	ICI	Non-ICI	Difference (per 1,000)	ICI	Non-ICI
1 year	1.95%	1.12%	1.73%	1.01%	7.2	17.1%	15.0%
2 years	3.05%	1.92%	2.63%	1.68%	9.5	21.0%	19.0%
3 years	3.97%	2.63%	3.34%	2.24%	11.0	23.8%	22.0%
5 years	5.50%	3.93%	4.47%	3.23%	12.5	27.4%	26.4%

Part B. Restricted mean survival time, free of glomerular disease

Time horizon (τ)	ICI (days)	Non-ICI (days)	Difference (days, 95% CI)
1 year	361.0	362.8	−1.72 (−2.08 to −1.36)
2 years	716.7	722.1	−5.42 (−6.43 to −4.41)
3 years	1,068.8	1,078.9	−10.05 (−11.93 to −8.17)
5 years	1,764.4	1,784.7	−20.28 (−24.63 to −15.93)

Part A. Death was modelled as a competing event using the Aalen–Johansen estimator. Cause-specific cumulative hazards for glomerular disease and for death were obtained from the corresponding Kaplan–Meier estimates in the matched cohort, and the cumulative incidence function was computed as the integral of the event-free survival function against the cause-specific hazard for glomerular disease. Because all-cause mortality was almost identical in the two cohorts, the competing risk attenuates the cumulative incidence in both arms by a similar factor (0.814 in the immune checkpoint inhibitor cohort and 0.820 in the comparator cohort at 5 years). The corresponding subdistribution hazard ratio is therefore approximately 1.48, compared with a cause-specific hazard ratio of 1.492, and the conclusion is unchanged. At 5 years the absolute difference in cumulative incidence is 1.24 percentage points, corresponding to 12.5 excess cases per 1,000 patients treated and a number needed to harm of approximately 80.

Part B. The proportional hazards assumption was violated for the primary outcome, so restricted mean survival time is reported as a supporting measure that does not require that assumption. Restricted mean survival time is the area under the survival curve up to the horizon τ and is interpreted as the average time free of glomerular disease within that horizon. Confidence intervals were obtained by integrating the pointwise standard error of the survival function and are conservative.

Table S20. Non-overlapping interval analysis of the primary outcome

Interval	ICI cumulative hazard increment	Non-ICI cumulative hazard increment	Interval hazard ratio
Day 1–500	0.0245	0.0146	1.68
Day 501–1000	0.0139	0.0100	1.39
Day 1001–1500	0.0106	0.0100	1.05
Day 1501–2000	0.0100	0.0085	1.17
Day 2001–3000	0.0091	0.0128	0.72

Each interval counts only the hazard accrued within that interval, among patients still under observation at its start; the intervals are therefore non-overlapping and are not cumulative landmark analyses. Interval hazard ratios were computed as the ratio of the increments in the cause-specific cumulative hazard, estimated from the Kaplan–Meier curves of the matched cohort. The excess hazard associated with immune checkpoint inhibitor therapy is concentrated in the first 500 days, and beyond approximately 1,000 days the interval point estimates approach unity. This pattern explains the apparent discrepancy between the hazard ratio and the cumulative risk ratio: the cumulative risk ratio converges towards the null over time because it accumulates person-time during which the two cohorts no longer differ, whereas the hazard ratio compares instantaneous rates among patients still at risk and is not diluted in the same way.

Methods S1. Additional methodological details

Study design and data source

This study used a target trial emulation framework to compare the risk of glomerular disease between patients with cancer treated with immune checkpoint inhibitors and those treated with conventional antineoplastic therapy. Data were obtained from the TriNetX US Collaborative Network, a federated network of de-identified electronic health records; 74 healthcare organisations were queried and 74 responded. The study period spanned 1 January 2014 to 1 March 2025, and the index eligibility window ended on 1 March 2024 so that every patient had at least 12 months of potential follow-up. Median follow-up was 764 days (interquartile range 1,111) in the immune checkpoint inhibitor cohort and 692 days (interquartile range 1,257) in the comparator cohort.

Cohort selection and exposure

Patients were required to have at least 6 months of continuous data before the index date so that baseline characteristics could be ascertained and pre-existing glomerular disease excluded. The index date was the first administration of the index therapy occurring within the index eligibility window; the date constraint was applied to the index event definition itself rather than only to the cohort-qualifying criteria, so that patients could not qualify for a cohort on the basis of treatment occurring after their assigned time origin. The requirement for repeated administrations was applied symmetrically to both cohorts.

Propensity score matching

A 1:1 greedy nearest-neighbour algorithm without replacement was used. The propensity score was estimated by multivariable logistic regression incorporating forty covariates spanning demographics, malignancy type, comorbidities, concomitant medications and baseline kidney function (Table S7). The caliper was 0.2 times the standard deviation of the logit of the propensity score. Standardised mean differences below 0.10 were considered to indicate adequate balance, and all covariates met this criterion after matching. Variables not entered into the model, including secondary malignant neoplasm, calendar year of index, baseline proteinuria and viral hepatitis, were assessed for post-matching balance.

Outcome ascertainment

Outcomes were identified from ICD-10-CM diagnosis codes, laboratory values and procedure codes as specified in Tables S4 to S6. For every outcome, patients with that outcome recorded before the start of the time window were excluded from the analysis of that outcome, so that all estimates refer to incident events. Because the codes identify clinically recognised diagnoses rather than biopsy-confirmed disease, a specificity gradient was examined by comparing the six-code composite with its most biologically specific component (N00 and N01) and with nephrotic syndrome alone (N04).

Statistical analysis

Kaplan–Meier estimates were compared by log-rank test and hazard ratios were estimated by Cox proportional hazards regression with robust standard errors accounting for the matched-pair structure. The proportional hazards assumption was assessed for every outcome; where it was violated, the hazard ratio is reported as a time-averaged effect and is supplemented by restricted mean survival time and by a non-overlapping interval analysis (Tables S19 and S20). Death was additionally modelled as a competing event using the Aalen–Johansen estimator, and the resulting cumulative incidence functions and absolute risk differences are reported in Table S19. E-values were calculated for the primary estimate and its lower confidence limit to quantify the strength of unmeasured confounding that would be required to explain the association.

Subgroup analyses

Subgroup estimates were obtained within strata of the matched cohort. Heterogeneity across the strata of each domain was tested by Cochran Q on the log hazard ratio scale, with one test per domain and Benjamini–Hochberg adjustment across domains; pairwise contrasts are post-hoc (Table S18). Subgroup analyses were not prespecified in a statistical analysis plan and matching was not repeated within strata, so all subgroup findings are reported as exploratory and hypothesis-generating.

Sensitivity analyses

Four sensitivity analyses were performed: an on-treatment analysis requiring at least six administrations within the first six months with a day-180 landmark (Table S13); exclusion of patients receiving nephrotoxic chemotherapy (Table S14);

exclusion of immune checkpoint inhibitor recipients who subsequently received nephrotoxic chemotherapy (Table S15); and a competing-risk analysis treating death as a competing event (Table S19).

Control outcomes

Three negative control outcomes with no plausible causal relationship to immune checkpoint inhibitor therapy were analysed to detect residual confounding, and two positive control outcomes representing established immune-related adverse events were analysed to demonstrate that the analytic pipeline detects true immune-related events (Table S16). In addition, the intensity of renal monitoring was compared between cohorts to assess differential detection (Table S17).

Limitations

Outcomes were defined by diagnosis codes rather than by histopathological adjudication, and kidney biopsy results, urine microscopy, quantitative proteinuria and serological testing are not uniformly available in the network, so individual diagnoses could not be validated. Cancer stage, dosing regimens and specific chemotherapy protocols were unavailable. Residual confounding by unmeasured variables cannot be excluded despite matching on forty covariates. Although the requirement for repeated administrations was applied symmetrically and a landmark analysis was performed, this design feature cannot be fully eliminated as a source of selection bias in a database without protocol-level treatment information.

Abbreviations

ACE, angiotensin-converting enzyme; AKI, acute kidney injury; ARB, angiotensin receptor blocker; ATC, Anatomical Therapeutic Chemical classification; BH, Benjamini–Hochberg; CI, confidence interval; CKD, chronic kidney disease; CPT, Current Procedural Terminology; CTLA-4, cytotoxic T-lymphocyte–associated protein 4; eGFR, estimated glomerular filtration rate; EHR, electronic health record; ESRD, end-stage renal disease; HR, hazard ratio; ICD-10-CM, International Classification of Diseases, Tenth Revision, Clinical Modification; ICI, immune checkpoint inhibitor; LAG-3, lymphocyte-activation gene 3; LOINC, Logical Observation Identifiers Names and Codes; MDRD, Modification of Diet in Renal Disease; NSAID, non-steroidal anti-inflammatory drug; PD-1, programmed cell death protein 1; PD-L1, programmed death-ligand 1; PH, proportional hazards; PSM, propensity score matching; RMST, restricted mean survival time; RR, risk ratio; RxNorm CUI, RxNorm concept unique identifier; SD, standard deviation; SMD, standardised mean difference.
